# α/β-Peptides as Nanomolar Triggers of Lipid Raft-Mediated Endocytosis through GM1 Ganglioside Recognition

**DOI:** 10.3390/pharmaceutics14030580

**Published:** 2022-03-07

**Authors:** Anasztázia Hetényi, Enikő Szabó, Norbert Imre, Kaushik Nath Bhaumik, Attila Tököli, Tamás Füzesi, Réka Hollandi, Peter Horvath, Ágnes Czibula, Éva Monostori, Mária A. Deli, Tamás A. Martinek

**Affiliations:** 1 Department of Medical Chemistry, University of Szeged, Dóm Tér 8, 6720 Szeged, Hungary; hetenyi.anasztazia@med.u-szeged.hu (A.H.); imrenorbert21@gmail.com (N.I.); kaushik.nb@pharm.u-szeged.hu (K.N.B.); attila.tokoli@gmail.com (A.T.); tamasfuzesi99@gmail.com (T.F.); 2 Institute of Genetics, Biological Research Centre, Temesvári krt. 62, 6726 Szeged, Hungary; szabo.eniko@brc.hu (E.S.); monostori.eva@brc.hu (É.M.); 3 Institute of Biophysics, Biological Research Centre, Temesvári krt. 62, 6726 Szeged, Hungary; hollandi.reka@brc.hu (R.H.); horvath.peter@brc.hu (P.H.); 4 Synthetic and Systems Biology Unit, Biological Research Centre, Temesvári krt. 62, 6726 Szeged, Hungary; deli@brc.hu

**Keywords:** cell delivery, glycan recognition, alpha-beta peptide, endocytosis, immunoglobulin

## Abstract

Cell delivery of therapeutic macromolecules and nanoparticles is a critical drug development challenge. Translocation through lipid raft-mediated endocytic mechanisms is being sought, as it can avoid rapid lysosomal degradation. Here, we present a set of short α/β-peptide tags with high affinity to the lipid raft-associated ganglioside GM1. These sequences induce effective internalization of the attached immunoglobulin cargo. The structural requirements of the GM1-peptide interaction are presented, and the importance of the membrane components are shown. The results contribute to the development of a receptor-based cell delivery platform.

## 1. Introduction

The efficient translocation of macromolecular cargoes and nanoparticles through the mammalian cell membrane is an important challenge in modern drug development [1,2,3]. Endocytic routes avoiding rapid lysosomal degradation are of interest because these facilitate endosomal escape and delivery to organelles or cytosol with limited cargo decomposition [4,5]. Triggering internalization at the lipid rafts is an emerging approach because this gateway generates endosomes that rarely fuse with lysosomes [6,7,8]. Lipid raft-mediated endocytosis can facilitate endosomal escape for the internalized cargoes [9,10]. Often, an abundance of different ganglioside molecules decorates the extracellular surface of the lipid rafts, on which pattern-specific recognition induces the desired endocytosis [11,12]. Ganglioside GM1 is the major receptor for the Cholera toxin, which utilizes this pathway [13,14]. In a recent study, we showed that a pentapeptide tag (WYKYW) could bind ganglioside GM1 with high affinity and thereby internalize an IgG complex (580 kDa) at low nanomolar extracellular concentrations [15]. The endocytotic pathways were tested with inhibitor experiments, demonstrating that lipid raft-mediated internalization is the major pathway for this process. The involvement of GM1 in the mechanism was supported by Cholera toxin co-localization and an inhibition experiment with the GM1 binder lectin galectin-1. Cell lines expressing different amounts of cell surface GM1 displayed internalization efficiency in accordance with their GM1 content. Inducing endocytosis with WYKYW avoided lysosomes and retained the functional structure of the cargo, which justifies further investigations.

Using various foldamers as cell-penetrating agents can offer efficient translocation of cargoes while being less susceptible to hydrolysis and the immune system [16,17,18,19,20]. Specifically, incorporating β-amino acids can lead to tuned interactions with the target molecule, which is beneficial to bioavailability [21]. Prior work determined the required sugar moieties on gangliosides for high-affinity binding with the WYKYW sequence [15]. Removal of the sialic acid or the terminal disaccharide moieties caused substantial affinity loss. In this work, our goal was to explore the structural requirements of the WYKYW– GM1 ganglioside interaction for the peptidic partner to help rational designs for expanding this family of sequences for cell delivery. Establishing a pharmacophore model of the ganglioside–peptide interaction appeared feasible in a structure-affinity relationship approach. To meet this challenge, we aimed to determine the effects of various amino acid replacements in the parent sequence, including side-chain removal (Ala-scan), stereochemical inversion (D-scan), and backbone homologation (β^3^-scan). In addition, the role of terminal tryptophan residues was investigated with the help of spectroscopic methods. The structure-affinity relationship analysis yielded improved unnatural peptides, efficiently internalizing large protein cargoes.

## 2. Materials and Methods

*Peptide Synthesis and Purification*: Peptide amides were synthesized with (7-azabenzotriazol-1-yl)tetramethyluronium hexafluorophosphate as a coupling agent (HATU) on TentaGel R RAM resin. (Sigma-Aldrich, Budapest, Hungary, product code: 86359) Coupling was carried out with a three times-equivalent excess of amino acid at room temperature for 3 h. The peptide cleavage from the resin was performed with TFA/water/d,l-dithiothreitol/triisopropylsilane (90:5:2.5:2.5), which was then precipitated in ice-cold diethyl ether. The resin was washed with acetic acid and water. The raw products were subsequently filtered and lyophilized. Peptide purification was performed using RP–HPLC with a C18 column. The HPLC eluents were 0.1% TFA in water and 0.1% TFA in ACN. Analytical RP–HPLC and ESI–MS measurements confirmed the purity of the peptides [22].

*Isothermal Titration Calorimetry*: ITC was performed using a MicroCal VP-ITC microcalorimeter at 35 °C. The monosialoganglioside GM1 was obtained from Biosynth-Carbosynth (Bratislava, Slovakia, product code: OG03918, C_73_H_131_N_3_O_31_·xNa, HPLC purity > 95%). The n-dodecylphosphocholine (DPC) was obtained from Avanti (Alabaster, Alabama, product code: 850336, C_17_H_38_NO_4_P, purity > 99%). In individual titrations, 15 µL of solution containing GM1:DPC 1:5 was injected into the ligand solution in the cell from a computer-controlled 300 µL microsyringe at time intervals of 300 s. The GM1:DPC micelle mixture was prepared in the same pH 7.2 phosphate buffer as the ligand in the cell. The concentration of the ligand in the cell was 15 µM. The concentration of GM1 in the syringe was 300 µM. In control experiments, GM1:DPC was titrated into the cell containing buffer without ligand. To rule out lipid clustering, control measurements were performed with pure GM1 in the syringe, which yielded a marked decrease in ΔH values, indicating the GM1:DPC interaction in the micelles (Figure S6). No aggregation of the micelles was observed after titration, which rules out any secondary inter-particle effects induced by ligand binding. We note that such an uncontrolled aggregation phenomenon would have been detrimental to the very sensitive ITC detection. Our finding is in agreement with the literature results that mixed micelles of GM1 and DPC are readily formed [23]. The experiments were repeated twice. The experimental data were fitted to one-binding-site or two-independent-site models with adjustable parameters of ΔH_b1_, K_d1_, n_1_, and ΔH_b2_, K_d2_, n_2_, respectively. Background subtraction and spline baseline correction was applied prior to the application of a generalized reduced gradient nonlinear least-squares procedure. The inert counter ions and residual solvent in the peptide, protein, and lipid samples normally caused residual heat during mixing, which was corrected with a constant term as an additional parameter in the model. Errors were estimated by using jack-knife resampling [22].

*Tryptophan fluorescence blue-shift measurements:* Fluorescence experiments were carried out at room temperature, in 20 mM PBS (pH 7.4) with a Hitachi F-2500 fluorescence spectrophotometer (PMT voltage: 700 V, response time: 0.08 s). Tryptophan was excited at the wavelength of 295 nm. Emission spectra were recorded in the range 300 to 400 nm. The excitation and emission bandwidths were set to 5 nm. Peptides were measured alone at a concentration of 2.5 μM and with the addition of either 250 μM DPC micelles or 50 μM GM1:250 μM DPC bicelles. Control measurements in the absence of the peptide were subtracted from the corresponding data [22].

*Circular dichroism*: Circular dichroism measurements were carried out with a Jasco J-815 CD Spectrometer. Spectra were recorded using a thermally jacketed 1 mm quartz cuvette in the wavelength range 260 to 195 nm. The scan speed was 50 nm min^−1^ with 5 accumulations. Peptide concentration was 200 μM in 10 mM PBS (pH 7.4). The effect of the binding on the CD curve was measured after adding 100:500 μM GM1:DPC bicelles. A Julabo water thermostat controlled the temperature with an equilibration time of 10 min at each temperature. Solvent baseline subtraction was applied [22].

*Cell Culture*: HeLa cells were cultured in advanced MEM (Gibco^®^/Invitrogen Corporation, New York, NY, USA) supplemented with 10% fetal bovine serum (FBS, PAN-Biotech, Aidenbach, Germany). Penicillin–streptomycin (100 U mL^−1^, Gibco^®^/Invitrogen Corporation, New York, NY, USA) and 2 mM L-glutamine (Gibco^®^/Invitrogen Corporation, New York, NY, USA) was added to the medium. The cells were grown at 37 °C in a humidified incubator containing 5% CO_2_ [22].

*Preparation of Carrier–Protein Complexes*: A solution of the biotinylated peptide, biotinylated monoclonal mouse anti-human [24] antibody and unlabelled Neutravidin (NA) (ThermoFisher Scientific, Watham, MA, USA, product code: 31000) was mixed at a molar ratio of 3:1:1. The secondary Alexa Fluor 647-conjugated F(ab’)2-goat anti-mouse IgG (Gibco^®^/Invitrogen Corporation, New York, NY, USA, product code: 31006) was added to the solution subsequently at a molar ratio of 1:1 relative to the primary antibody. The dilution of this complex was set before adding to HeLa cells [22].

*Live Confocal Laser Scanning Microscopy*: HeLa cells were plated on six-chamber µ-Slides VI 0.4 (ibidi, Gräfelfing, Germany) for overnight culturing in MEM + 10% FBS at 1.25 × 10^4^ cells per cm^2^ (or 1.5 × 10^4^ cells per channel). After washing with PBS, the cells were incubated at 37 °C with the complexes in MEM + 1% FBS medium for the required time lengths, which was followed by washing with PBS. The cells were stained with 100 ng mL^−1^ Hoechst 33342 (Sigma-Aldrich St. Louis, MO, USA) in MEM medium for 30 min at 37 °C. Cell membranes were visualized after a 5 min treatment with FITC-labelled WGA lectin at 0.2 µg mL^−1^ at room temperature. The cells were incubated in Leibovitz’s L-15 medium (Gibco^®^/Invitrogen Corporation, New York, NY, USA) during microscopic analysis. Cell fluorescence was analyzed to observe the localization of the cargo, using a Leica SP5 AOBS confocal laser scanning microscope with a 405 nm UV diode (for Hoechst staining), a 488 nm argon laser line (for FITC staining), and a 633 HeNe laser line (for Alexa Fluor 647 staining). An appropriate spectral filter was used for each channel for the detection of the emissions [22].

*Image Analysis*: We used Mask R-CNN, a deep learning-based image segmentation platform for identifying cells and extracting their properties; U-Net, another deep learning approach; and CellProfiler software for feature extraction. Cell nuclei were identified on the basis of the Hoechst signal using a very heavily augmented training set of The Data Science Bowl 2018 competition. The augmentation was performed by learning image styles and generating synthetic images of similar types with Pix2Pix, a generative adversarial network (GAN) deep network. Subsequently, a Mask R-CNN network was trained, and individual nuclei were inferred. A similarly augmented image set was used to train a U-Net deep convolutional neural network, using the FITC-WGA lectin channel images and binary masks marking the cytoplasm as foreground. The trained U-Net network predicted the foreground pixels corresponding to cytoplasm. We approximated the cytoplasm with a watershed region propagation algorithm on the weighted sum image of the U-Net prediction and the FITC-WGA lectin channel. With the help of the detected objects (nucleus and cytoplasm) as masks, cellular features such as Alexa 647 intensity values, textural properties, and morphological descriptors were extracted. The integrated intensities of individual cells were used for the final statistical analysis [22].

*Protease assays*: Peptides were dissolved in 100 mM TRIS–HCl buffer (pH 8.0) containing 10 mM CaCl_2_ at 100 mM concentration. Chymotrypsin (Sigma-Aldrich, Budapest, Hungary) stock solution was 0.5 mg/mL in the same buffer. A total 500 mL peptide solution was measured in a reaction vessel and 50 mL protease stock was added. Samples of 50 mL were taken after 2, 6, 25 and 60 min. The samples were diluted into 500 mL 5% TFA aliquots. The samples were then injected into HPLC–MS using a Phenomenex C18-XB Peptide column (250 × 4.6 mm, 3.6 mm). For the experiments with trypsin, the same method was used, except for the buffer, which did not contain any CaCl_2_.

## 3. Results

First, we performed an Ala-scan on the WYKYW sequence, substituting each of the five amino acids one by one, and measured their affinity to GM1:DPC bicelles using isothermal titration calorimetry (ITC) [25]. Dissociation constants (Table 1, Figures S1–S3) revealed that any side chain removal from the original sequence was detrimental to binding. This finding suggests that WYKYW is a minimal sequence because all side chains were essential in stabilizing the GM1–WYKYW complex. Modification of the central Lys led to the largest affinity decrease, indicating the role of the cationic function. Removal of the neighboring Tyr residues had the second largest effect on the affinity. From the Ala-scan, we can conclude that the core tripeptidic sequence (YKY) has a dominant contribution to stabilizing the GM1–WYKYW complex.

High-affinity WYKYW–GM1 interactions project a precise fit between the two partners, which requires a specific stereochemical pattern along the peptidic chain. We tested this with a systematic D-amino acid substitution. Again, we found that any configuration change resulted in a marked decrease in the affinities. The central Lys residue was the most sensitive. Interestingly, inversion of Tyr4 and Trp5 yielded lower affinities than the Ala replacement. This observation suggests that stereochemical inversion not only disrupted the interaction, but that the modified side-chain orientation exerted an extra destabilization effect. We concluded that no change in the amino acid configuration is tolerable, which strongly supports that the molecular recognition between GM1 and WYKYW requires a specific peptide geometry. To further test the sensitivity of the interaction to the backbone homologation, we carried out β^3^-amino acid replacements in the sequence. Strikingly, the incorporation of the β^3^-h-Trp at the N-terminal yielded improved affinity. The GM1–WYKYW complex displayed good tolerance to the backbone homologation, except for the central β^3^-h-Lys residue. This pattern in the structure-affinity relationship corroborates that the central YKY motif and its fine geometry play crucial roles in ganglioside recognition. The protease resistance of the homologated sequences was also measured. As expected, the single β^3^-amino acid replacements at the central residues provided increased stability against proteases (Table S1).

**Figure 1:**
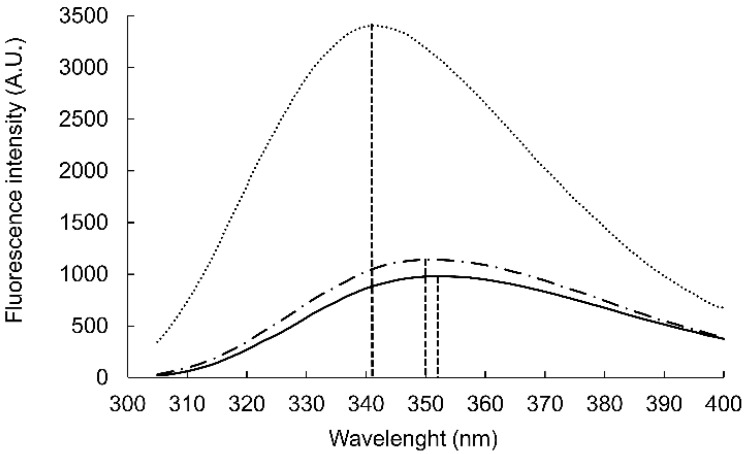
The blue-shift of the tryptophan fluorescence emission in interaction with GM1:DPC micelles. Emission spectra obtained for peptide WYKYW alone (black, 2.5 µM), WYKYW + DPC (DPC 250 µM, dash-dotted), and WYKYW + GM1:DPC (+GM1:DPC 50:250 µM, dotted).

While the structure-affinity relationship study revealed the importance of the central YKY motif, the Trp residues at the termini contribute a large interaction surface, pointing to the role of hydrophobic interactions. We hypothesized that these residues immerse into the fatty acid region of the membrane [25] and thereby stabilize the WYKYW–GM1 interaction over the membrane surface. The Trp fluorescence maximum wavelength depends on the environment; relocation of the aromatic side chain from an aqueous solvent to a hydrophobic environment causes a blue shift [26]. The Trp fluorescence was measured in the presence of DPC micelles and GM1:dodecylphosphocholine (DPC) bicelles (Figure 1). WYKYW alone had an emission maximum of 350 nm. The addition of DPC micelles caused a minor blue shift and intensity increase. In contrast, GM1:DPC bicelles induced a blue shift of the emission maximum to 341 nm, accompanied by a marked intensity increase. This observation suggests that Trp moved to a hydrophobic environment in the membrane and became partially shielded from the water. The extent of the shift was consistent with a relative permittivity of 7, which suggests that the Trp side chains were neither in the aqueous medium nor completely buried in the hydrophobic interior near the headgroups of the amphipathic lipids [27]. This location is in good accordance with the requirement that the segment YKY is close to the carbohydrate moiety of GM1.

**Figure 2:**
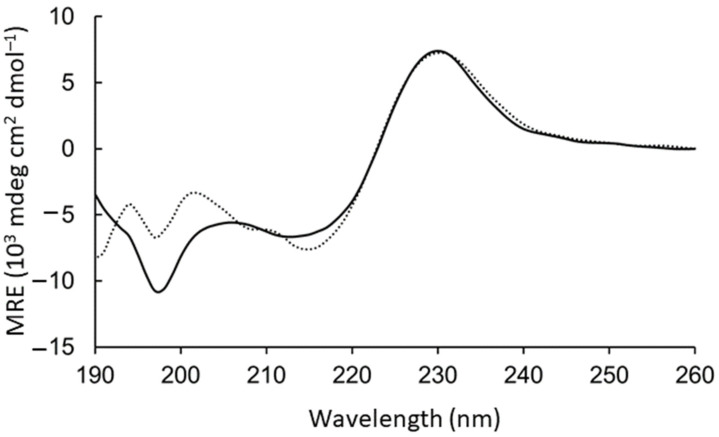
CD spectra measured for peptide WYKYW alone (black, 200 µM) and after adding 100:500 µM GM1:DPC bicelles (dotted).

The aromatic side chains dominated the circular dichroism spectrum, preventing direct conclusions about the backbone geometry. However, intensity loss at 198 nm was observed upon adding the GM1:DPC bicelles (Figure 2). To test the contribution to this region from the aromatic rings, WAKAW and AYKYA were synthesized and measured (Figure S4 long-dashed). Thus, the intensive peak at 198 nm could be assigned to Trp–Trp face-to-edge interactions in solution [28,29], which is lost in the GM1-bound form.

**Figure 3:**
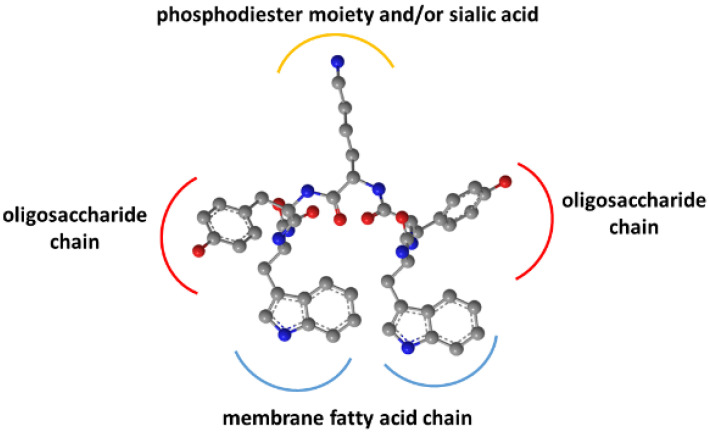
Pharmacophore hypothesis generated from the SAR and Trp-fluorescence data.

As previously shown, binding did not occur with the pentasaccharide moiety of GM1 without the hydrophobic sphingosine and fatty acid parts in the membrane [15]. Removal of the sialic acid also strongly decreased affinity. Together with the Trp residues’ membrane insertion, these findings suggested an interaction model for WYKYW–GM1 interactions (Figure 3).

The model rationalizes the importance of the stereochemical configuration. It also explains the backbone homologation tolerance at the termini. The position of Trp side chains in the dynamic membrane is adaptive, whereas the side chain distances have to be more specific in the YKY segment to recognize the oligosaccharide moiety.

**Figure 4:**
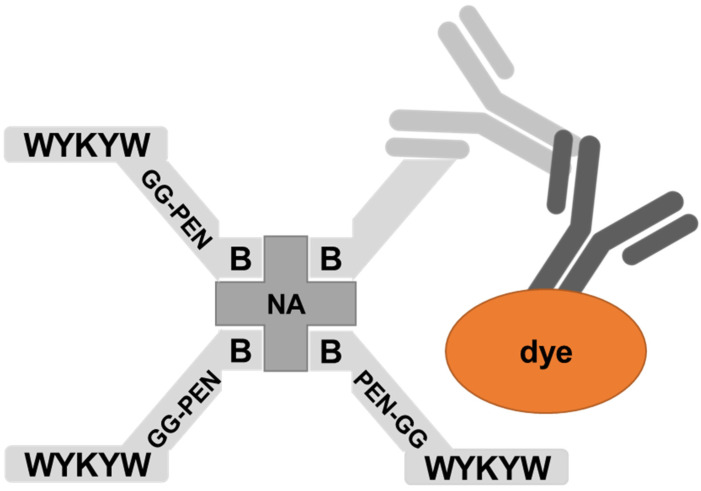
Schematic representation of the bottom-up designed modular carrier–hub–antibody cargo–secondary antibody–fluorescent-dye construct.

The nanomolar affinity to GM1 of the α/β-peptidic sequences projected their ability to trigger endocytosis. Therefore, we tested these carriers for protein internalization as previously described [30]. The peptides were attached to a Neutravidin hub through a biotinylated linker (Figure 4). The cargo was a protein complex containing a primary and a fluorescent secondary antibody. We chose penetratin as a linker sequence because it is unable to internalize the protein cargo by itself, but its cationic nature improves the solubility of the construct. Control experiments were run with the Neutravidin-antibody complex without carrier peptides.

**Figure 5:**
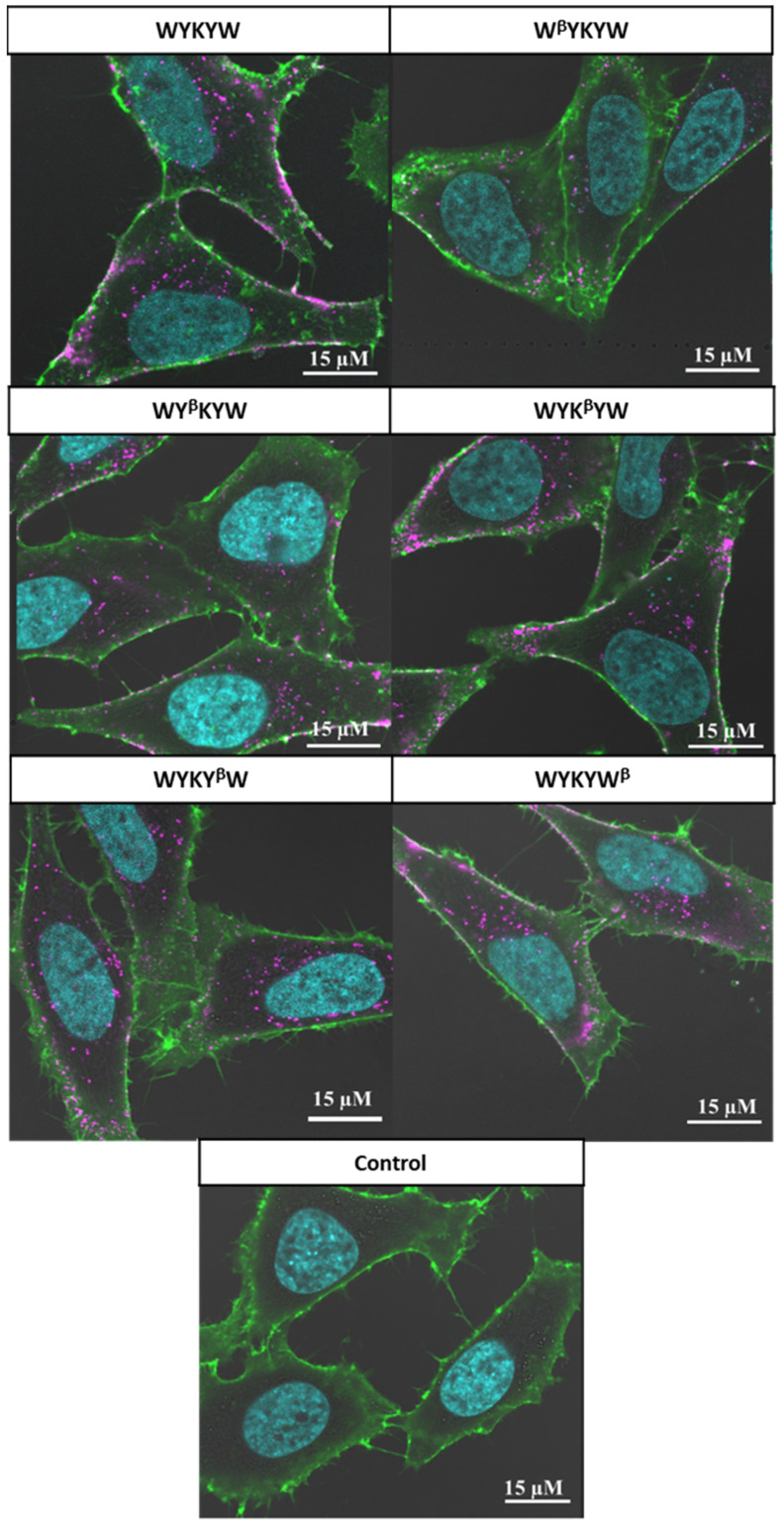
Delivery of the IgG cargo into HeLa cells, using the carrier WYKYW and its α,β-peptidic derivatives. The schematic representation of the carrier–cargo complex is given in Figure 4. Images are tagged by the carrier sequence measured (β denotes the corresponding β^3^-amino acid). Alexa Fluor 647-conjugated secondary antibody is indicated in magenta; green staining defines cell membranes (WGA-FITC). Nuclei are indicated in cyan. The carrier–cargo complexes we applied at a concentration of 80 nM, and cells were incubated for 1 h. In the control measurement, cells were treated with IgG complex without the ganglioside-binding carrier peptides attached.

**Figure 6:**
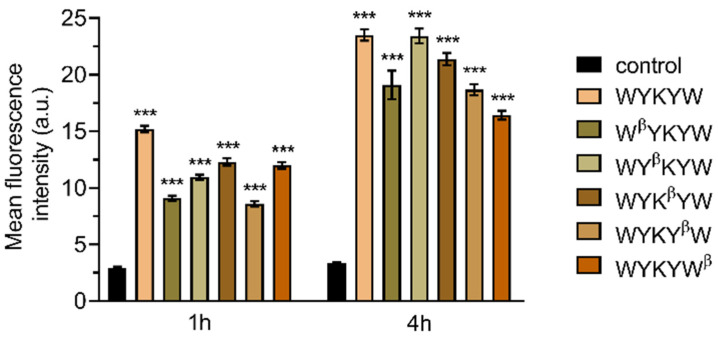
Artificial intelligence-aided quantitative analysis of the live CLSM images. HeLa cells were incubated for 1 or 4 h with the carrier–cargo complexes at 80 nM. At least 150 representative cells were then analyzed at each setup. The graph shows mean fluorescence intensity values ± standard error of the mean (SEM). Statistical analysis was performed using one-way analysis of variance (ANOVA) with Dunnett’s multiple comparison test, where each sample was compared to the control sample of the matching time point. *** *p* < 0.001.

Their internalization efficiency was monitored with CLSM (Figure 5). HeLa cells were incubated with the carrier–cargo complexes for 1 or 4 h at an extracellular concentration of 80 nM. The α/β-peptidic tags were able to induce endocytosis. We carried out an AI-based quantitation with the different analogues (Figure 6). Strikingly, no correlation was found with the GM1 binding affinities measured for the single carrier sequences. We hypothesized that the multiple presentations of the carrier on the complex increases avidity, thereby facilitating the endocytosis. To this end, we prepared the NA(biotinyl-Penetratin-WYK^β^YW)_4_ constructs, where the K_D_ of the monomers were 332 nM. ITC measurements showed a K_D_ of 38 nM for the multivalent construct (Figure S5), explaining the efficient internalization. The stoichiometry of this interaction was 1:1 relative to the concentration of the hub Neutravidin, in contrast to the 2:1 carrier:GM1 ratio. We note that Neutravidin is a tetrameric protein that can display two carrier peptides simultaneously toward the membrane. These findings strongly support the avidity-increasing effect of the multivalent presentation of the carrier.

## 4. Discussion

In summary, we found a sequence-dependent affinity for the WYKYW derivatives to the GM1 ganglioside. Contribution to the binding of each residue was demonstrated. We found that the stereochemical configuration pattern is crucial. The central YKY segment recognizes the oligosaccharide moiety, while the terminal Trp residues stabilize the complex by partial membrane insertion. We identified backbone-homologated analogues, which have affinities comparable with the parent sequence. We showed that the α/β-peptidic sequences internalize the multivalent carrier–IgG cargo complex with good efficiency with the help of an avidity increase.

## 5. Patents

A patent application has been accepted overlapping with the findings presented in this manuscript: WO2020245617.

## Figures and Tables

**Table 1:** Binding affinities (K_D_ [nM]) of the WYKYW analogues to ganglioside GM1. The binding stoichiometry (n_1_) was 0.5 in all cases. Superscripts indicate the corresponding peptide termini.

	^N^W	Y	K	Y	W^C^
original	23.8				
Ala-scan	n.f. ^[a]^	5755	10,467	1694	1060
D-scan	881	892	4523	3243	3926
β-scan	4.3	60	332	40	86

^[a]^ not fittable.

## Data Availability

All data supporting the findings of this study are available within the article and its supplemental information, or from the corresponding author upon reasonable request.

## Supplementary Materials

The following supporting information can be downloaded at: https://www.mdpi.com/article/10.3390/pharmaceutics14030580/s1, Figure S1: ITC detection of interactions of GM1 with Ala derivatives, Figure S2: ITC detection of interactions of GM1 with d-derivatives, Figure S3: ITC detection of interactions of GM1 with β-derivatives, Figure S4: CD spectrum of peptides, Figure S5: ITC detection of interactions of GM1 with derivatives, Figure S6: ITC enthalpograms of WYKYW–GM1, Table S1: Half-lives of compounds in protease assay with chymotrypsin, Table S2: Statistical Analysis, and Peptide characterization data.
