# Supplementary material for: α/β-Peptides as Nanomolar Triggers of Lipid Raft-Mediated Endocytosis through GM1 Ganglioside Recognition
Source: Pharmaceutics. 2022 Mar 7;14(3):580. doi: 10.3390/pharmaceutics14030580 (PMC8953856; doi:10.3390/pharmaceutics14030580)

Supporting Information

α/β-Peptides as nanomolar triggers of lipid raft-mediated endocytosis through GM1 ganglioside recognition

Anasztázia Hetényi [1,5], Enikő Szabó [2,5], Norbert Imre [1], Kaushik Nath Bhaumik [1], Attila Tököli [1], Tamás Füzesi [1], Réka Hollandi [3], Peter Horvath [3], Ágnes Czibula [2,*], Éva Monostori [2], Mária A. Deli [4] and Tamás A. Martinek [1,*]

1 Department of Medical Chemistry, University of Szeged, Dóm tér 8., 6720 Szeged, Hungary
2 Institute of Genetics, Biological Research Centre, Temesvári krt. 62., 6726 Szeged, Hungary
3 Institute of Biophysics, Biological Research Centre, Temesvári krt. 62., 6726 Szeged, Hungary
4 Synthetic and Systems Biology Unit, Biological Research Centre, Temesvári krt. 62., 6726 Szeged, Hungary
5 These authors contributed equally to this work
* Correspondence: martinek.tamas@med.u-szeged.hu, czibula.agnes@brc.hu

Table of contents

Figure S1. ITC detection of interactions of GM1 with *Ala*-derivatives. S2
Figure S2. ITC detection of interactions of GM1 with *d*-derivatives S2
Figure S3. ITC detection of interactions of GM1 with β-derivatives S2
Figure S4. CD spectrum of peptides S3
Figure S5. ITC detection of interactions of GM1 with carrier-NA conjugates S3
Figure S6. ITC enthalpograms of WYKYW-GM1 interaction with pure GM1 and GM1:DPC S3
Table S1. Half-lives of compounds in protease assay with chymotrypsin S4
Table S2. Statistical Analysis S4
Peptide characterization data S5

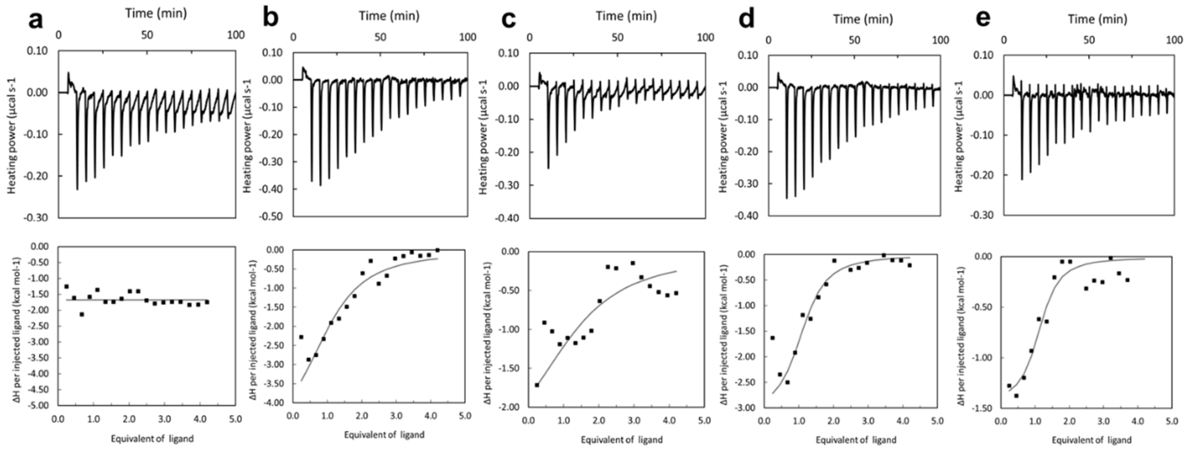

Figure S1. ITC detection of interactions of GM1 with *Ala* derivatives (a) AYKYW, (b) WAKYW, (c) WYAYW, (d) WYKAW and (e) WYKYA. Titrations were carried out with GM1:DPC 1:5 bicelles.

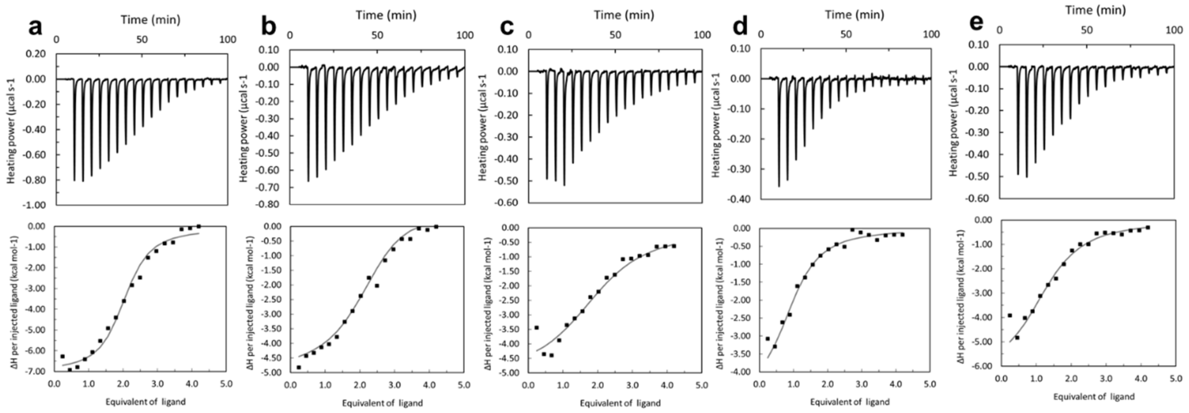

Figure S2. ITC detection of interactions of GM1 with *d* derivatives (a) wYKYW, (b) WyKYW, (c) WYkYW, (d) WYKyW and (e) WYKYw. Titrations were carried out with GM1:DPC 1:5 bicelles.

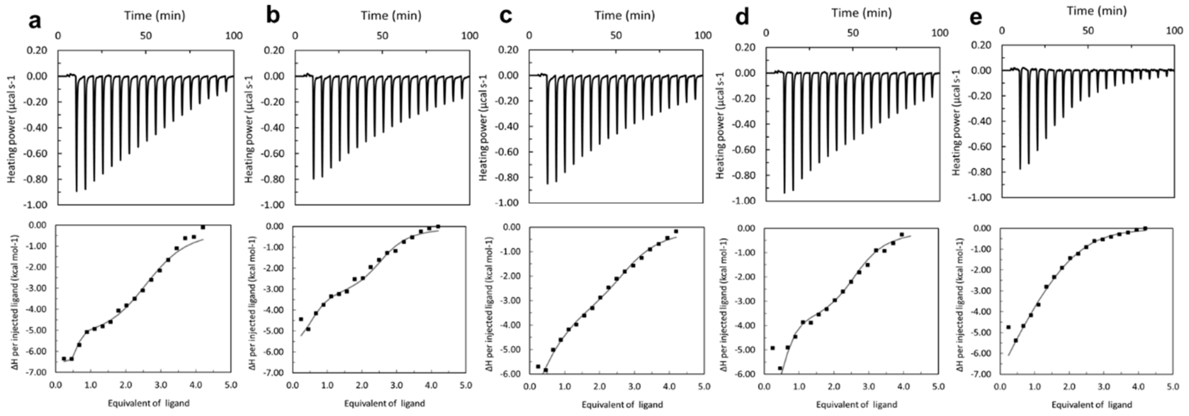

Figure S3. ITC detection of interactions of GM1 with β-derivatives (a) WβYKYW, (b) WYβKYW, (c) WYKβYW, (d) WYKYβW and (e) WYKYWβ. Titrations were carried out with GM1:DPC 1:5 bicelles.

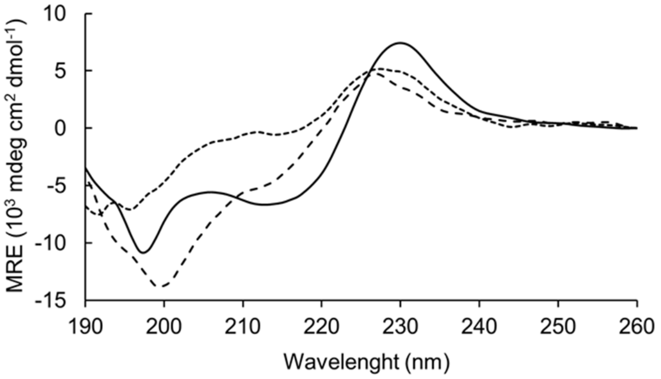

Figure S4. CD spectrum of peptides WYKYW (black), WAKAW (long dashed), and AYKYA (short dashed) (200 µM) at 303 K.

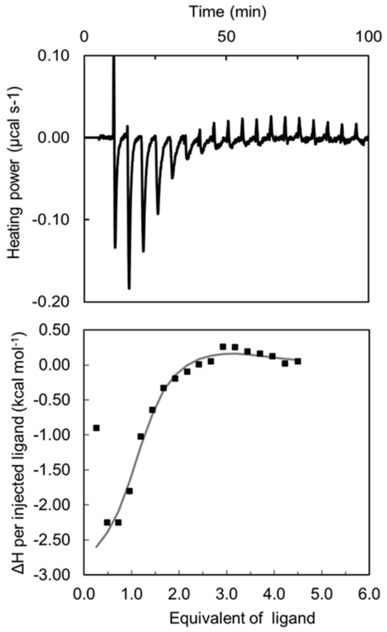

Figure S5. ITC detection of interactions of GM1 with carrier-NA conjugates. Enthalpograms of NA(biotinyl-Penetratin-WYK$^{\beta}$YW)$_4$ (n=1, K_D= 79 nM). Titrations were carried out with GM1:DPC 1:5 bicelles.

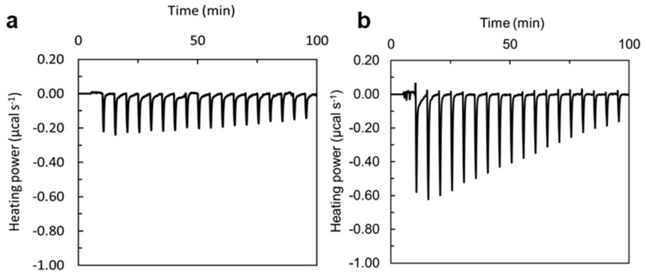

Figure S6. ITC enthalpogram obtained for WYKYW with pure GM1 micelles (a) and with GM1:DPC 1:5 bicelles (b).

Table S1. Half-lives of compounds in protease assay with chymotrypsin. In most cases, the peak belonging to the sequence was not detected at the first sampling (2 minutes), hence the half-life is < 24s with a 3% detection limit. * No degradation was detected during the experiment.

Sequence	Half-life (s)	
	Chymotrypsin	Trypsin
WYKYW	< 24	< 24
W^{β}YKYW	< 24	110
WY^{β}KYW	75	900
WYK^{β}YW	650	>> 24 h*
WYKY^{β}W	< 24	410
WYKYW^{β}	< 24	< 24

Statistical Analysis: Statistical analysis included one-way analysis of variance (ANOVA) with post hoc Tukey honestly significant difference test (**p* < 0.1; ***p* < 0.01; ****p* < 0.001; *****p* < 0.0001). The results of the statistical test are included in Table S2.

Table S2

**1 hour**	**Summary**	**Adjusted P Value**
WYKYW vs. W^{β}YKYW	****	<0,0001
WYKYW vs. WY^{β}KYW	****	<0,0001
WYKYW vs. WYK^{β}YW	*	0,0448
WYKYW vs. WYKY^{β}W	****	<0,0001
WYKYW vs. WYKYW^{β}	**	0,0099
W^{β}YKYW vs. WY^{β}KYW	ns	0,1567
W^{β}YKYW vs. WYK^{β}YW	***	0,0001
W^{β}YKYW vs. WYKY^{β}W	ns	>0,9999
W^{β}YKYW vs. WYKYW^{β}	***	0,0008
WY^{β}KYW vs. WYK^{β}YW	ns	0,5618
WY^{β}KYW vs. WYKY^{β}W	*	0,0141
WY^{β}KYW vs. WYKYW^{β}	ns	0,8793
WYK^{β}YW vs. WYKY^{β}W	****	<0,0001
WYK^{β}YW vs. WYKYW^{β}	ns	>0,9999
WYKY^{β}W vs. WYKYW^{β}	****	<0,0001

**4 hours**	**Summary**	**Adjusted P Value**
WYKYW vs. W^{β}YKYW	****	<0,0001
WYKYW vs. WY^{β}KYW	ns	0,1364
WYKYW vs. WYK^{β}YW	****	<0,0001
WYKYW vs. WYKY^{β}W	****	<0,0001
WYKYW vs. WYKYW^{β}	****	<0,0001
W^{β}YKYW vs. WY^{β}KYW	****	<0,0001
W^{β}YKYW vs. WYK^{β}YW	ns	0,0734
W^{β}YKYW vs. WYKY^{β}W	ns	>0,9999
W^{β}YKYW vs. WYKYW^{β}	**	0,0082
WY^{β}KYW vs. WYK^{β}YW	ns	0,1742
WY^{β}KYW vs. WYKY^{β}W	****	<0,0001
WY^{β}KYW vs. WYKYW^{β}	****	<0,0001
WYK^{β}YW vs. WYKY^{β}W	*	0,0248
WYK^{β}YW vs. WYKYW^{β}	****	<0,0001
WYKY^{β}W vs. WYKYW^{β}	ns	0,0891

Peptide characterization data

HPLC chromatograms and MS spectra for each sequence are shown below.

WYKYW (WYKYW-NH$_2$)

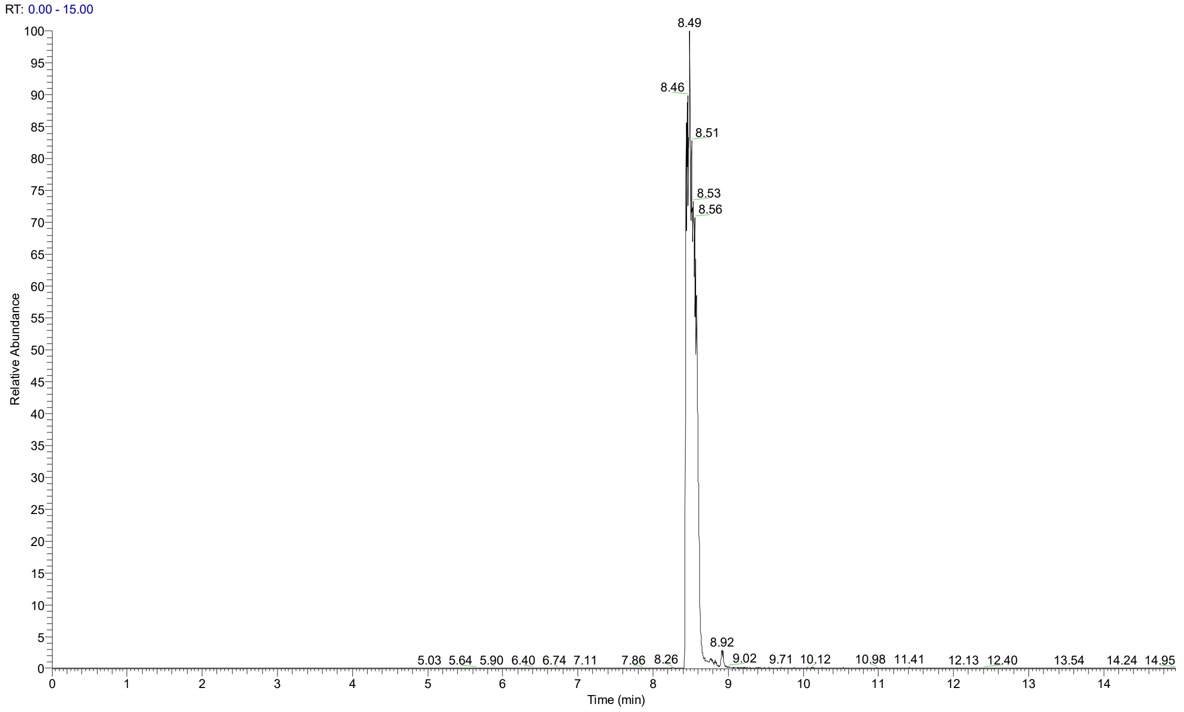

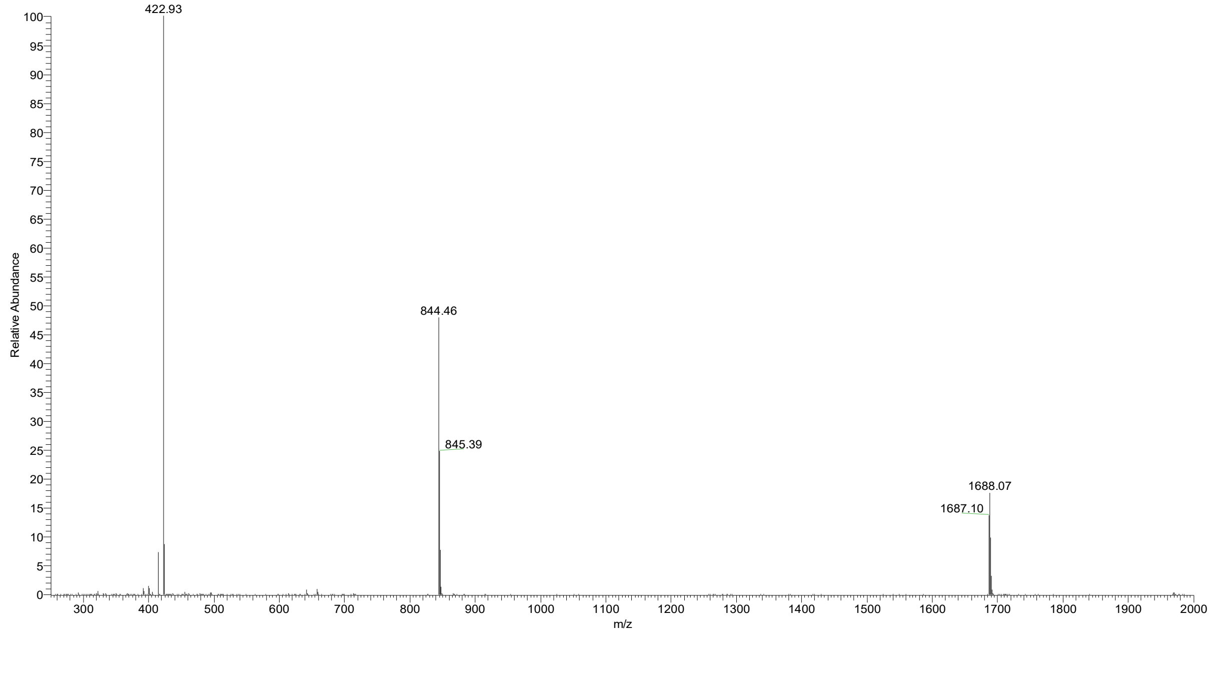

W$^{\beta}$YKYW

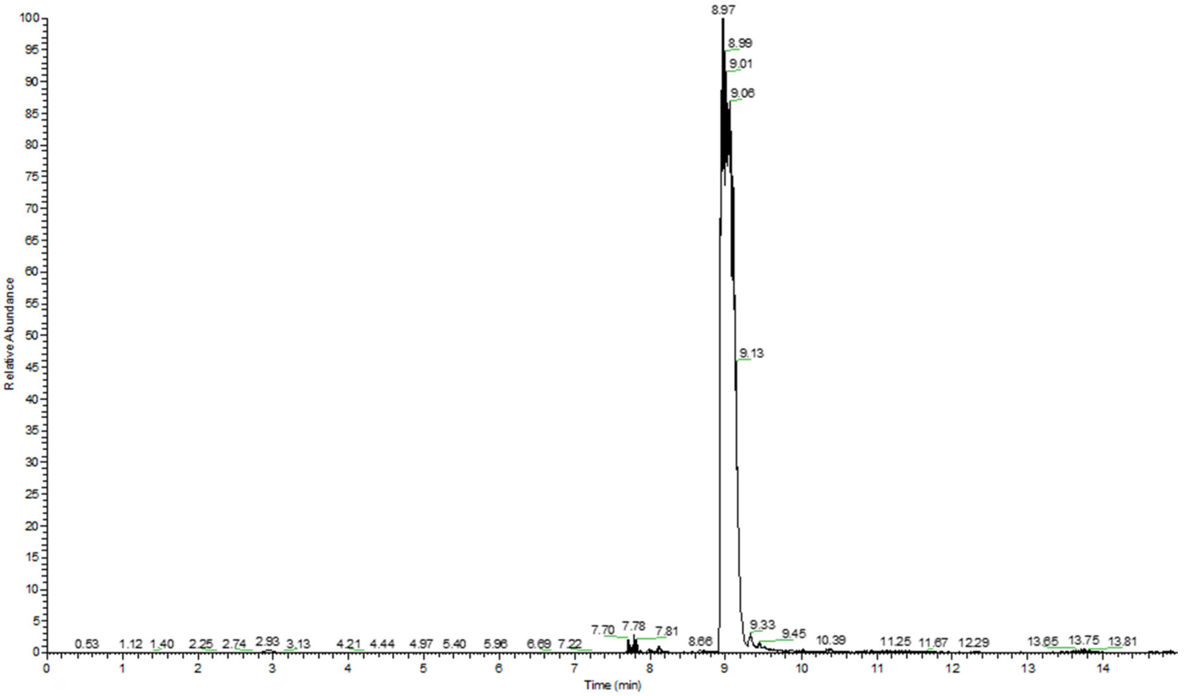

8.97
8.99
9.01
9.06
9.13
9.33
9.45
10.39
11.25
11.67
12.29
13.65
13.75
13.81
0.53
1.12
1.40
2.25
2.74
2.93
3.13
4.21
4.44
4.97
5.40
5.96
6.69
7.22
7.70
7.78
7.81
8.66
Relative Abundance
Time (min)

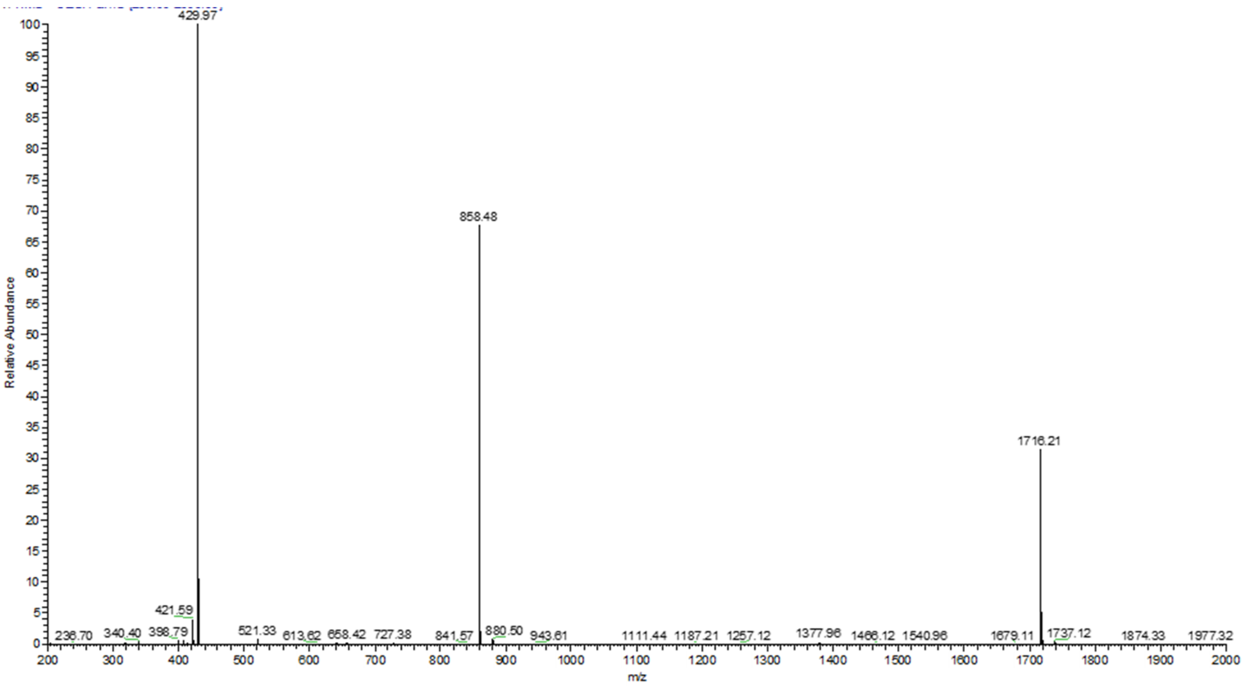

429.97
858.48
1716.21
421.59
236.70
340.40
398.79
521.33
613.62
658.42
727.38
841.57
880.50
943.61
1111.44
1187.21
1257.12
1377.96
1466.12
1540.96
1679.11
1737.12
1874.33
1977.32
Relative Abundance
m/z

WY$^{\beta}$KYW

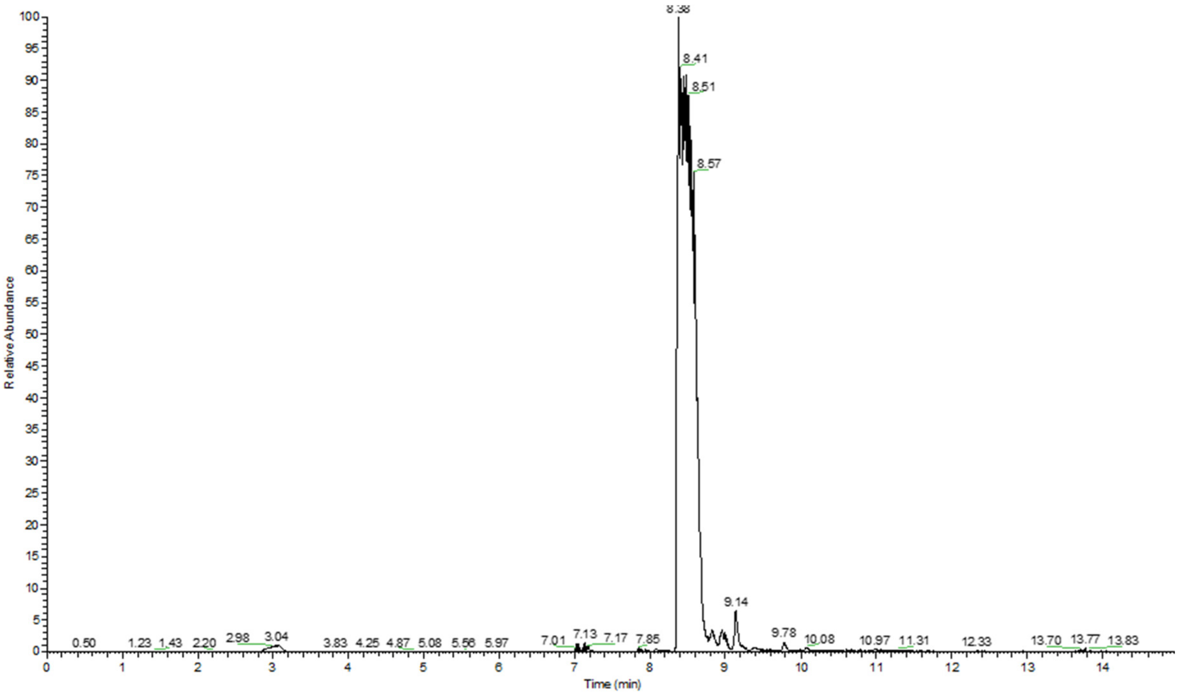

Relative Abundance
Time (min)
8.38
8.41
8.51
8.57
9.14
9.78

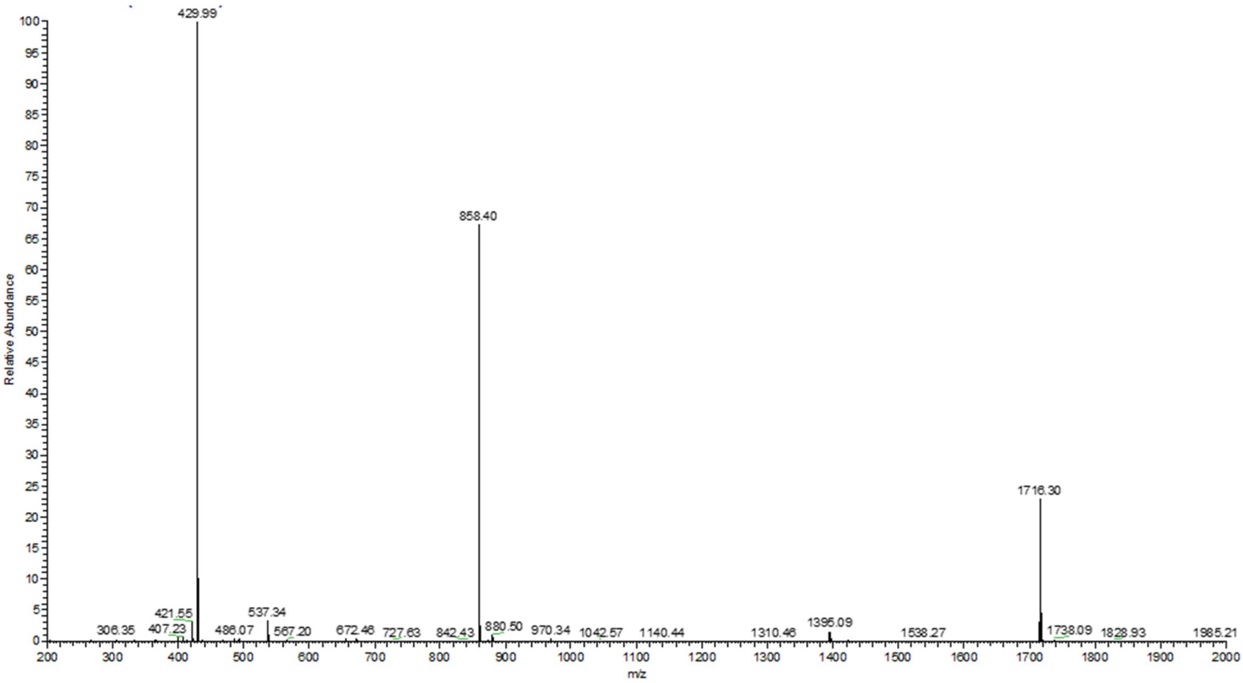

Relative Abundance
m/z
429.99
858.40
1716.30
537.34
421.55
1395.09

WYK$^{\beta}$YW

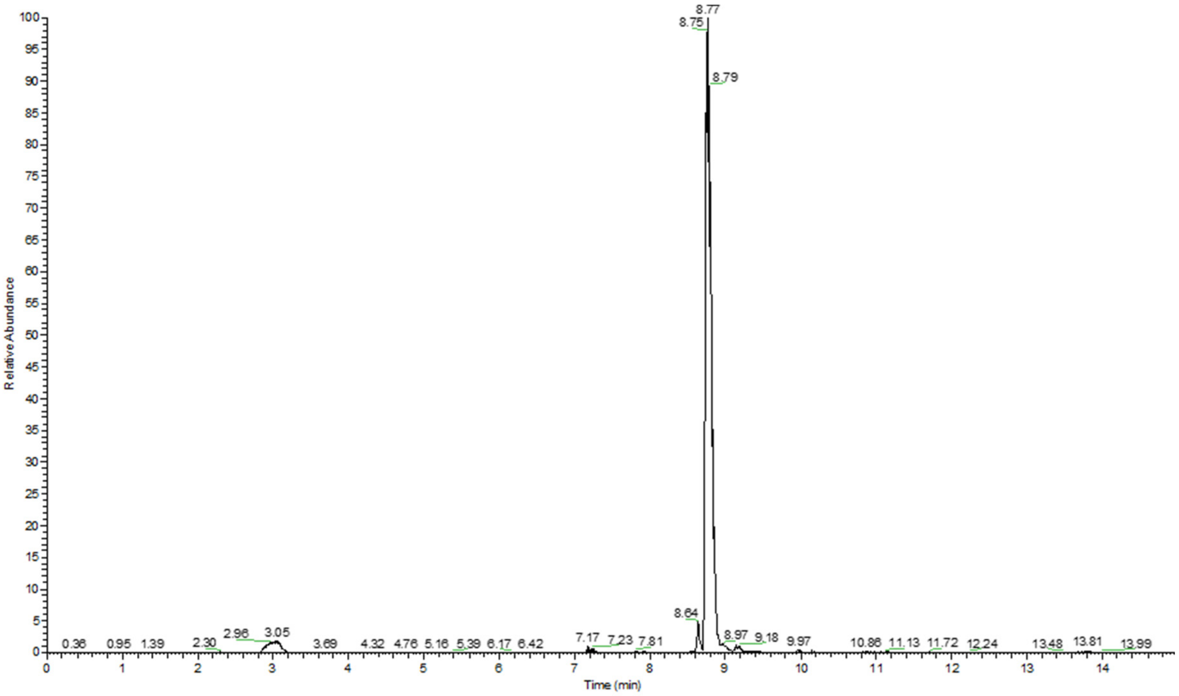

Relative Abundance
Time (min)
8.77
8.75
8.79
8.64
3.05

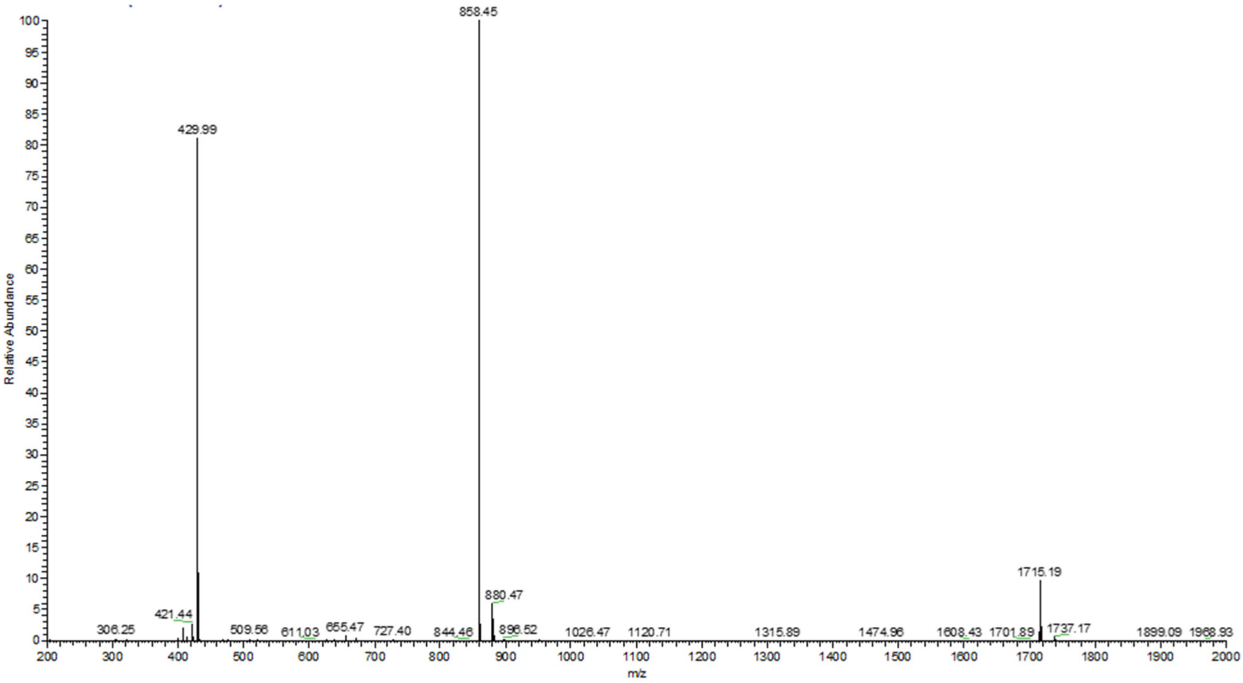

Relative Abundance
m/z
858.45
429.99
1715.19
880.47
421.44

WYKY$^{\beta}$W

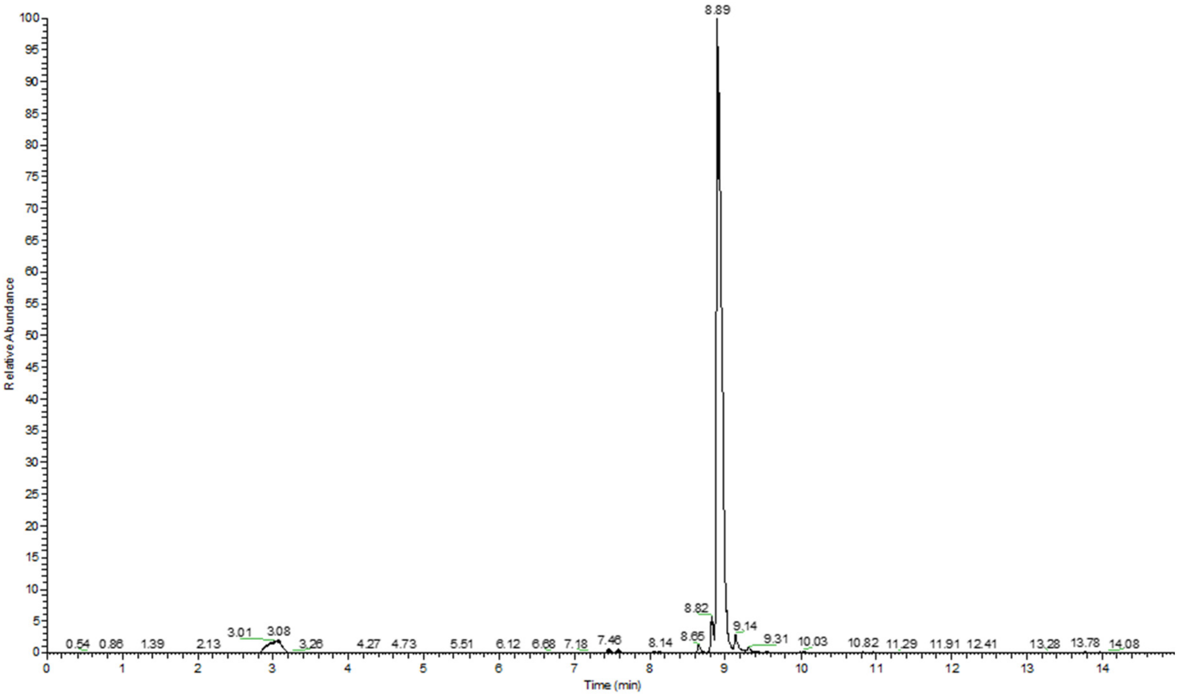
Relative Abundance
Time (min)
8.89
8.82
9.14
3.08

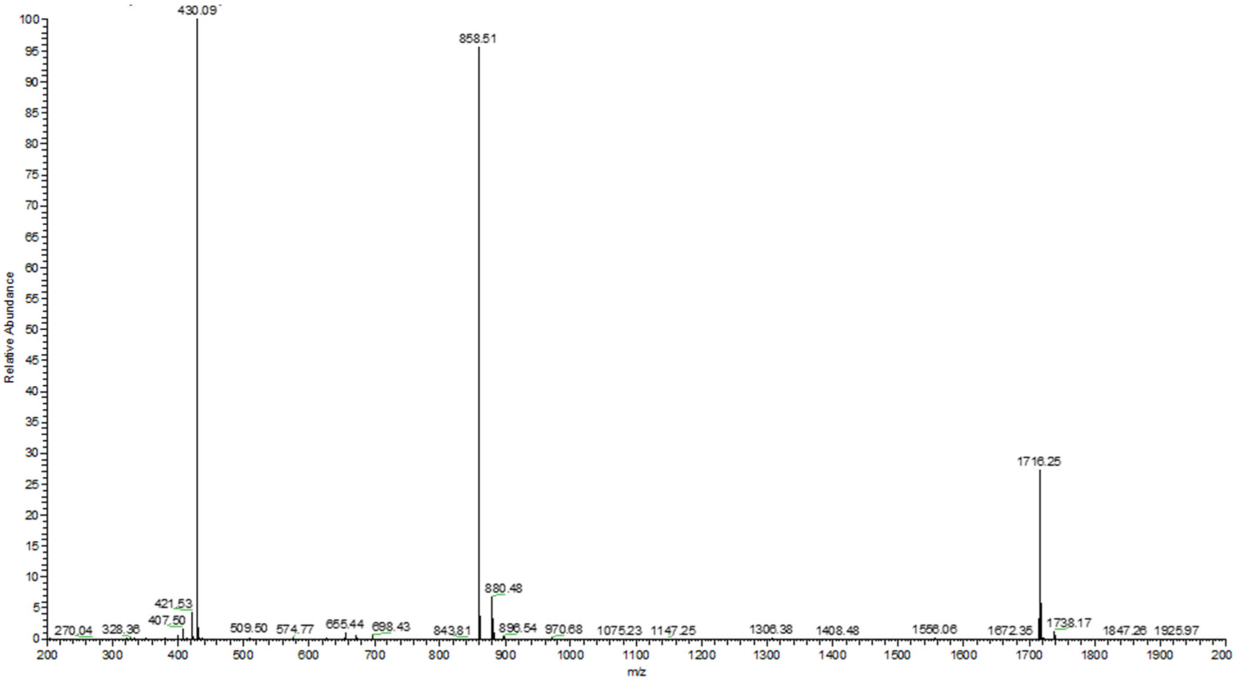
Relative Abundance
m/z
430.09
858.51
1716.25
880.48
421.53
1738.17

WYKYW$^{\beta}$

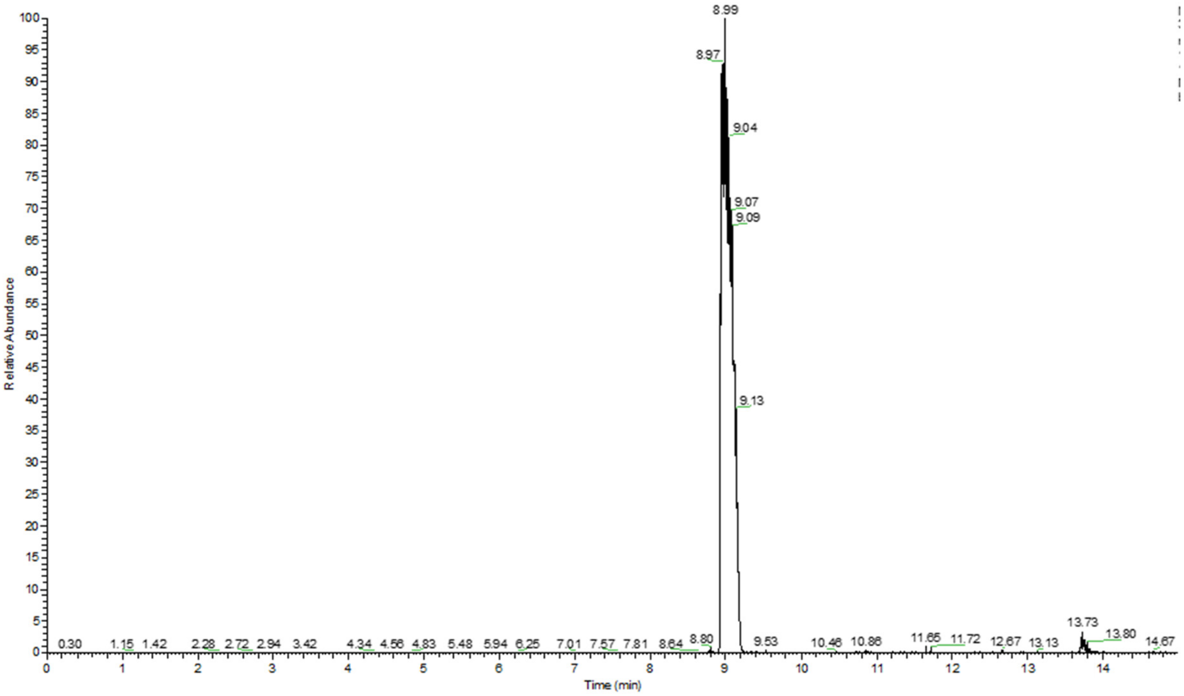
8.99
8.97
9.04
9.07
9.09
9.13
Relative Abundance
0.30 1.15 1.42 2.28 2.72 2.94 3.42 4.34 4.56 4.83 5.48 5.94 6.25 7.01 7.57 7.81 8.64 8.80 9.53 10.46 10.86 11.65 11.72 12.67 13.13 13.73 13.80 14.67
Time (min)

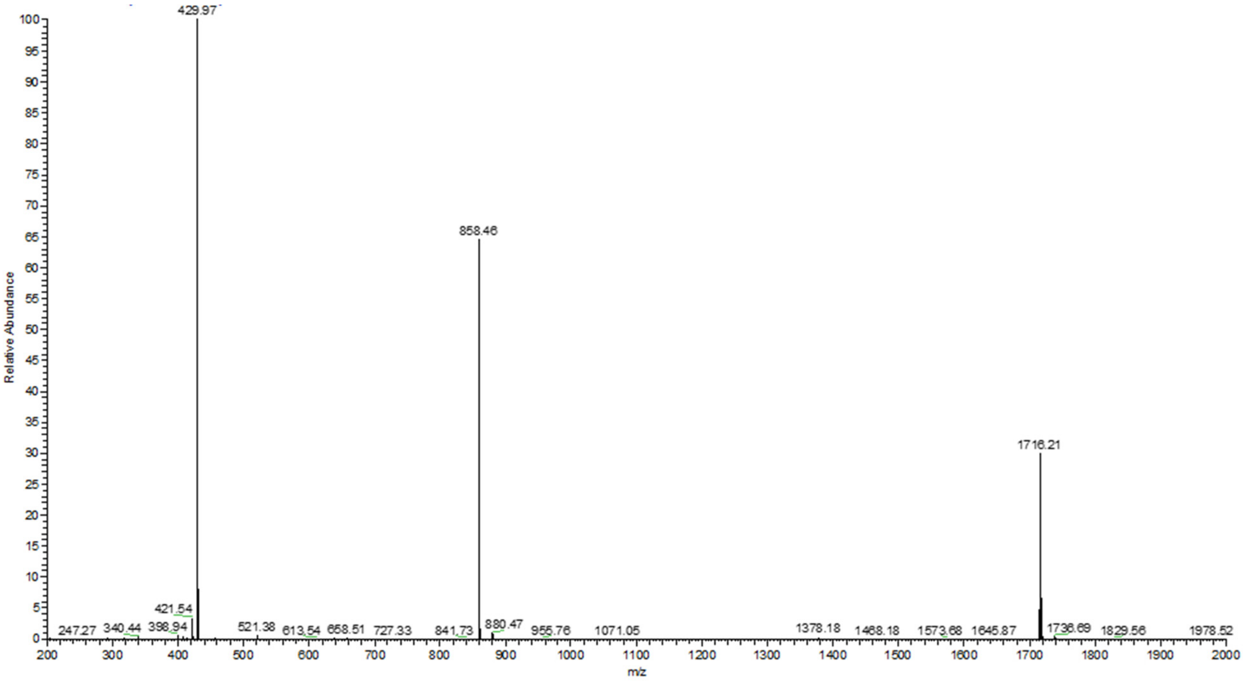
429.97
858.46
1716.21
Relative Abundance
247.27 340.44 398.94 421.54 521.38 613.54 658.51 727.33 841.73 880.47 955.76 1071.05 1378.18 1468.18 1573.68 1645.87 1736.69 1829.56 1978.52
m/z

AYKYW

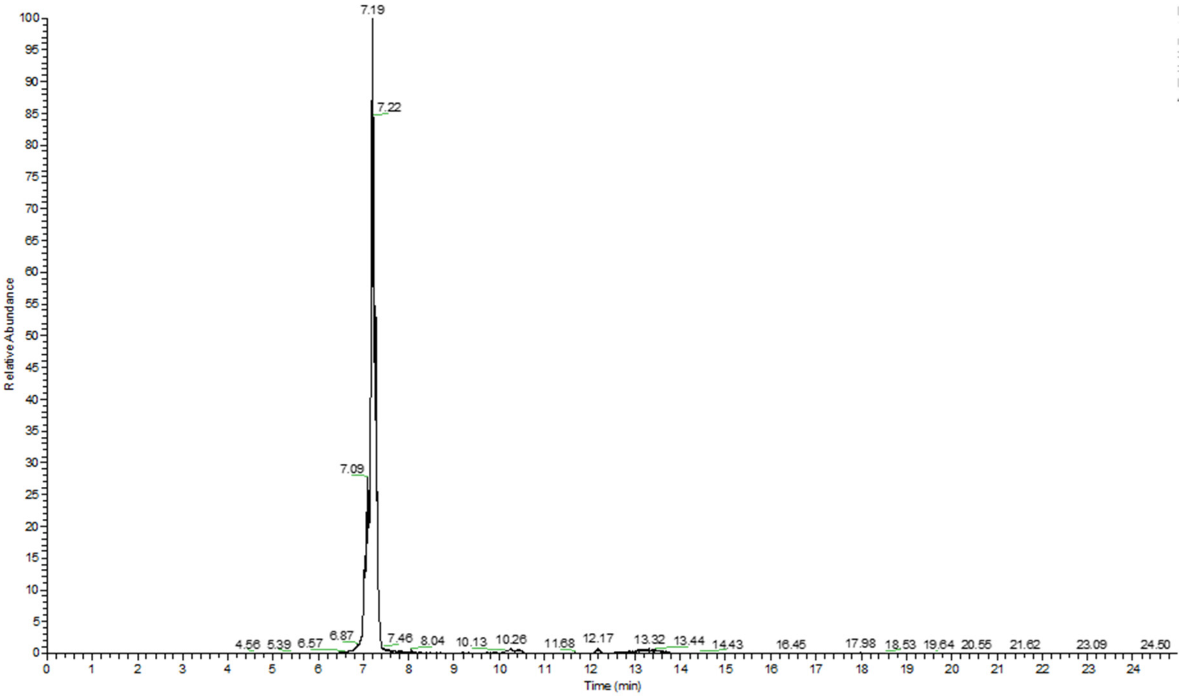

7.19
7.22
7.09
4.56
5.39
6.57
6.87
7.46
8.04
10.13
10.26
11.68
12.17
13.32
13.44
14.43
16.45
17.98
18.53
19.64
20.55
21.62
23.09
24.50
Relative Abundance
Time (min)

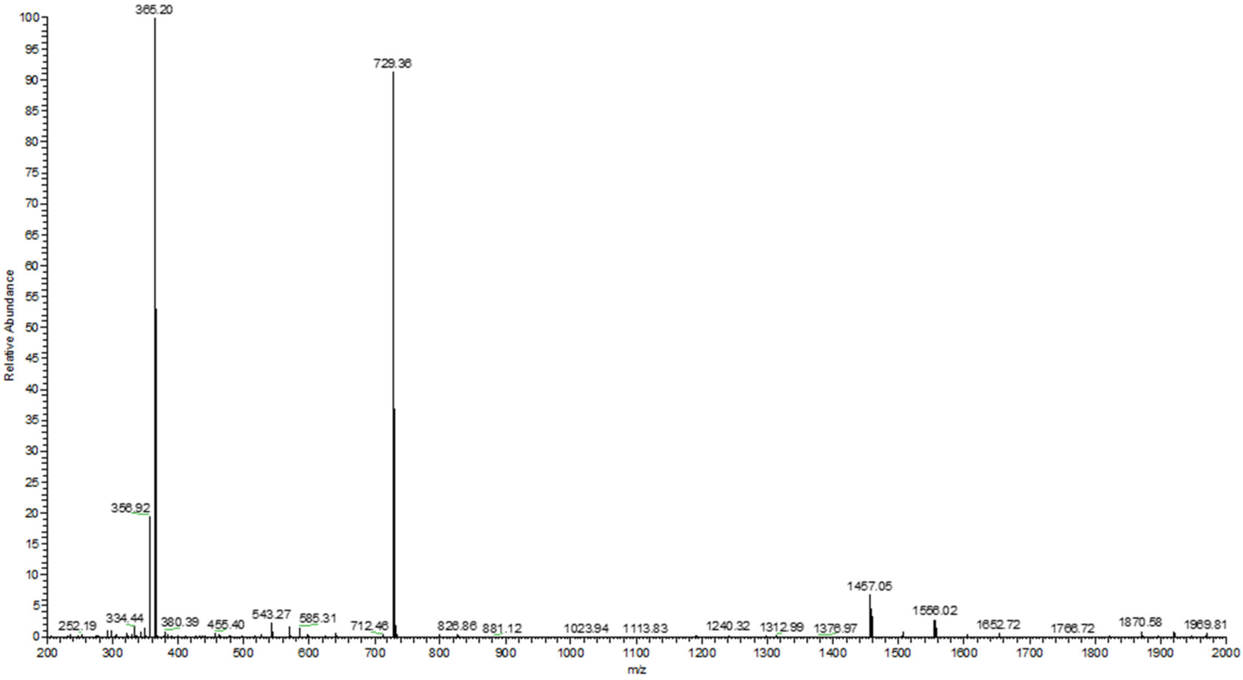

365.20
729.36
356.92
252.19
334.44
380.39
455.40
543.27
585.31
712.46
826.86
881.12
1023.94
1113.83
1240.32
1312.99
1376.97
1457.05
1556.02
1652.72
1766.72
1870.58
1969.81
Relative Abundance
m/z

WAKYW

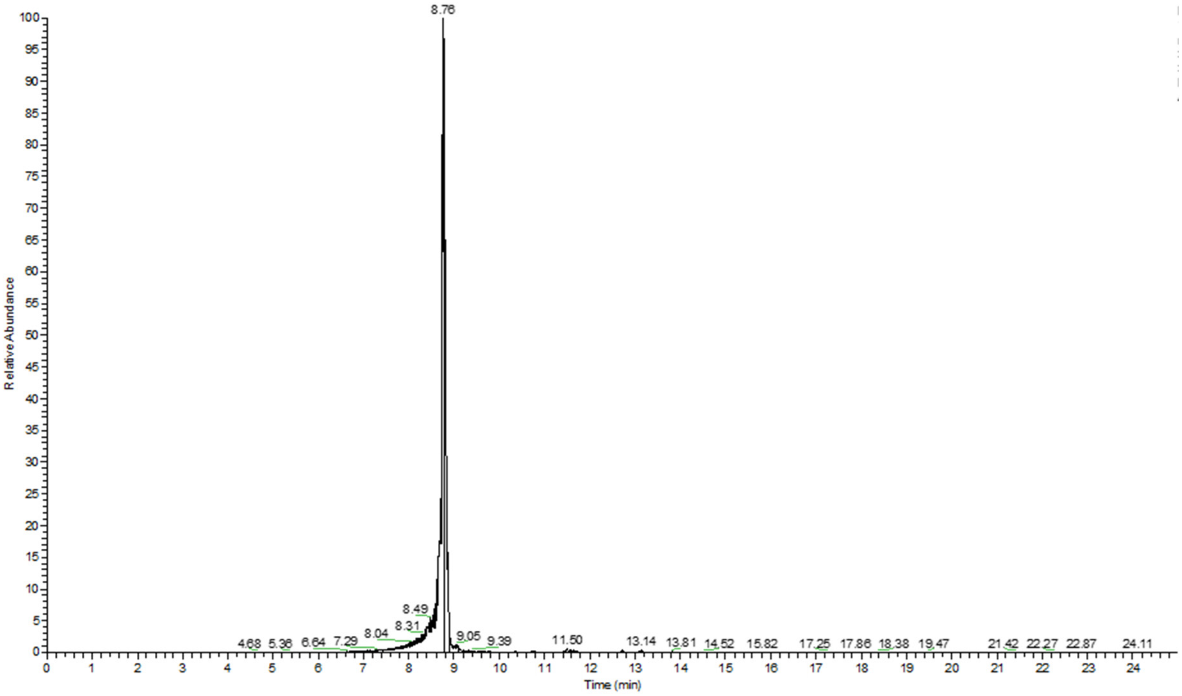

8.76
Relative Abundance
Time (min)

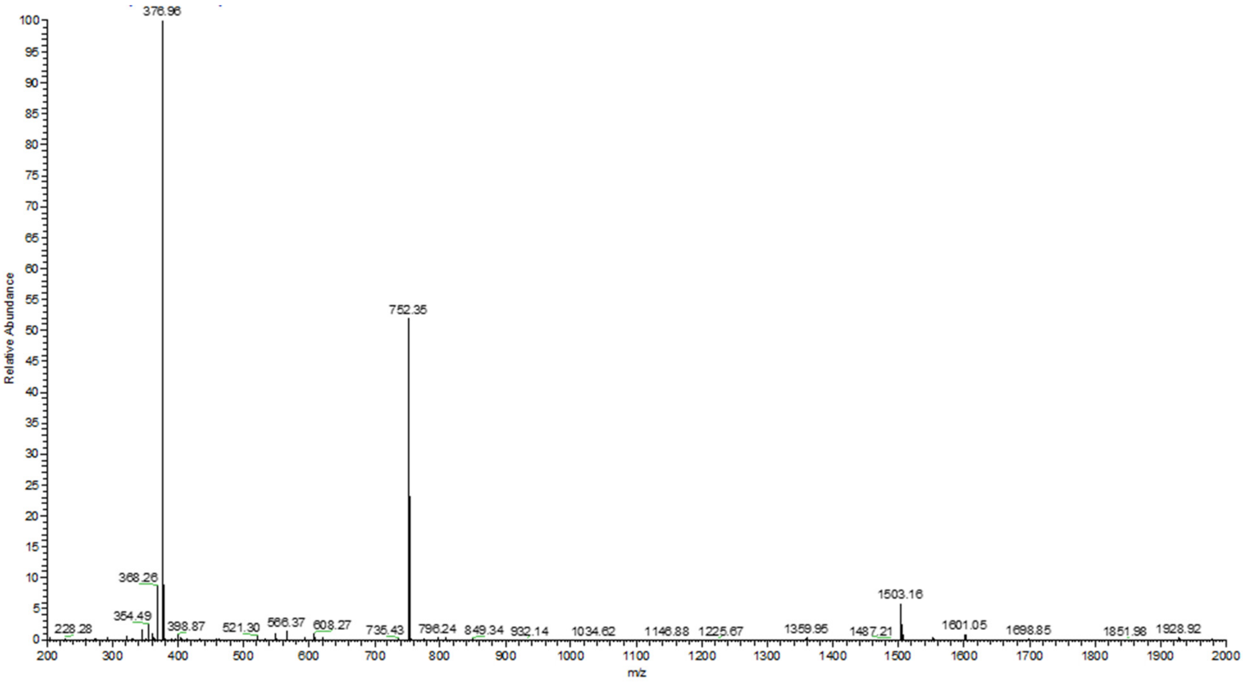

376.96
752.35
1503.16
Relative Abundance
m/z

WYAYW

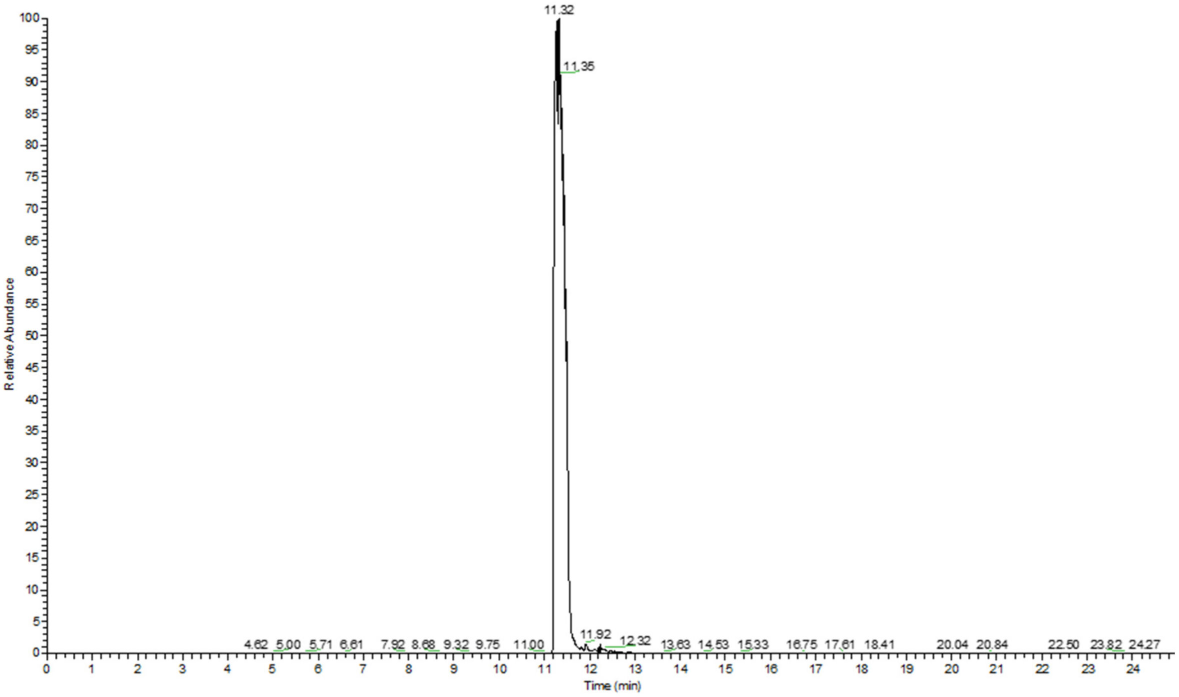

11.32
11.35
11.92
12.32
4.62 5.00 5.71 6.61 7.92 8.68 9.32 9.75 11.00
13.63 14.53 15.33 16.75 17.61 18.41 20.04 20.84 22.50 23.82 24.27
Relative Abundance
Time (min)

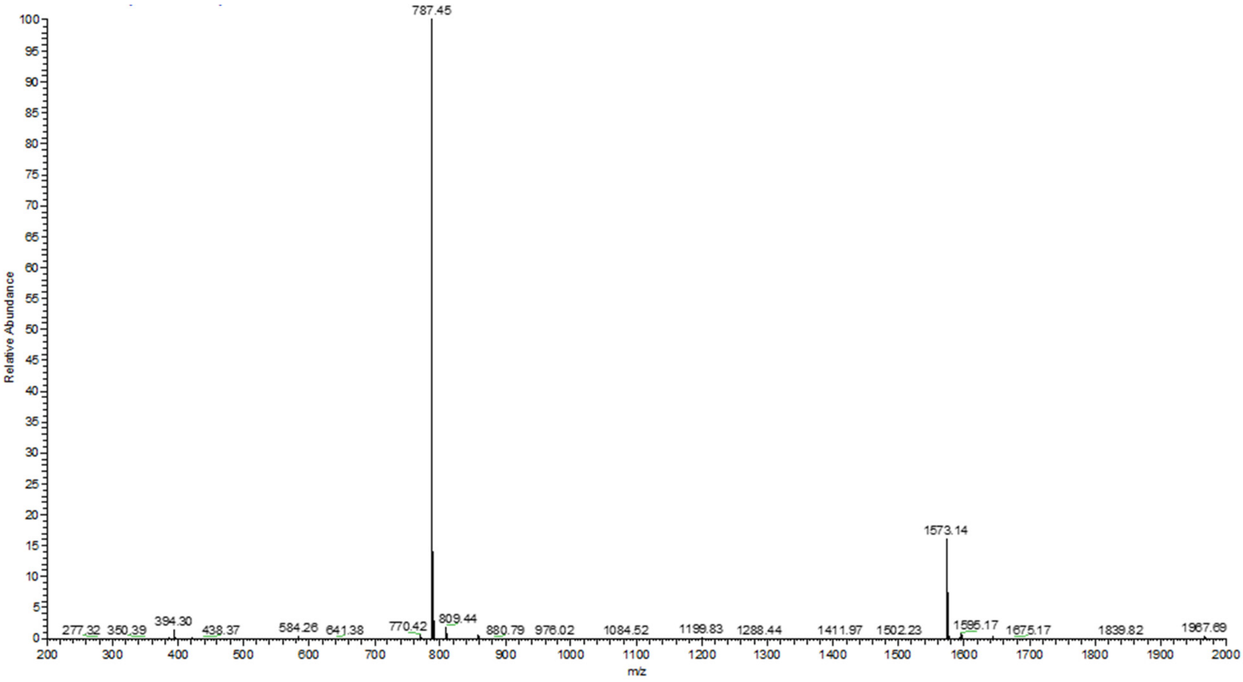

787.45
1573.14
277.32 350.39 394.30 438.37 584.26 641.38 770.42 809.44 880.79 976.02 1084.52 1199.83 1288.44 1411.97 1502.23 1595.17 1675.17 1839.82 1967.69
Relative Abundance
m/z

WYKAW

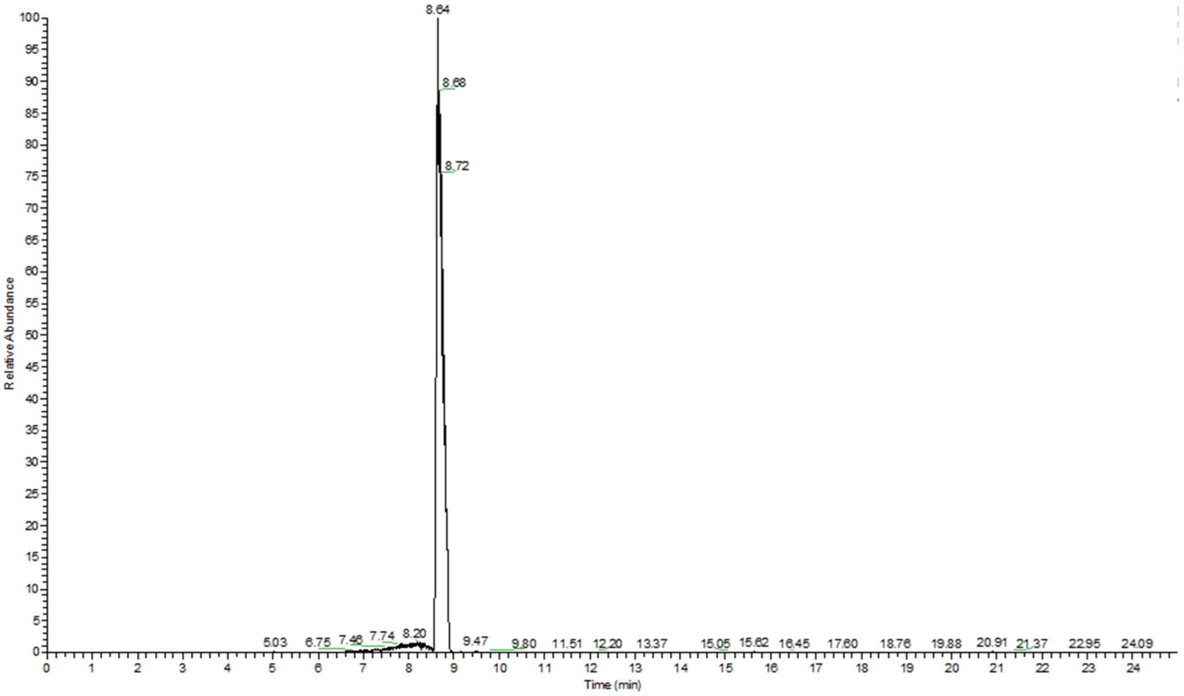

8.64
8.68
8.72
5.03
6.75
7.46
7.74
8.20
9.47
9.80
11.51
12.20
13.37
15.05
15.62
16.45
17.60
18.76
19.88
20.91
21.37
22.95
24.09
Relative Abundance
Time (min)

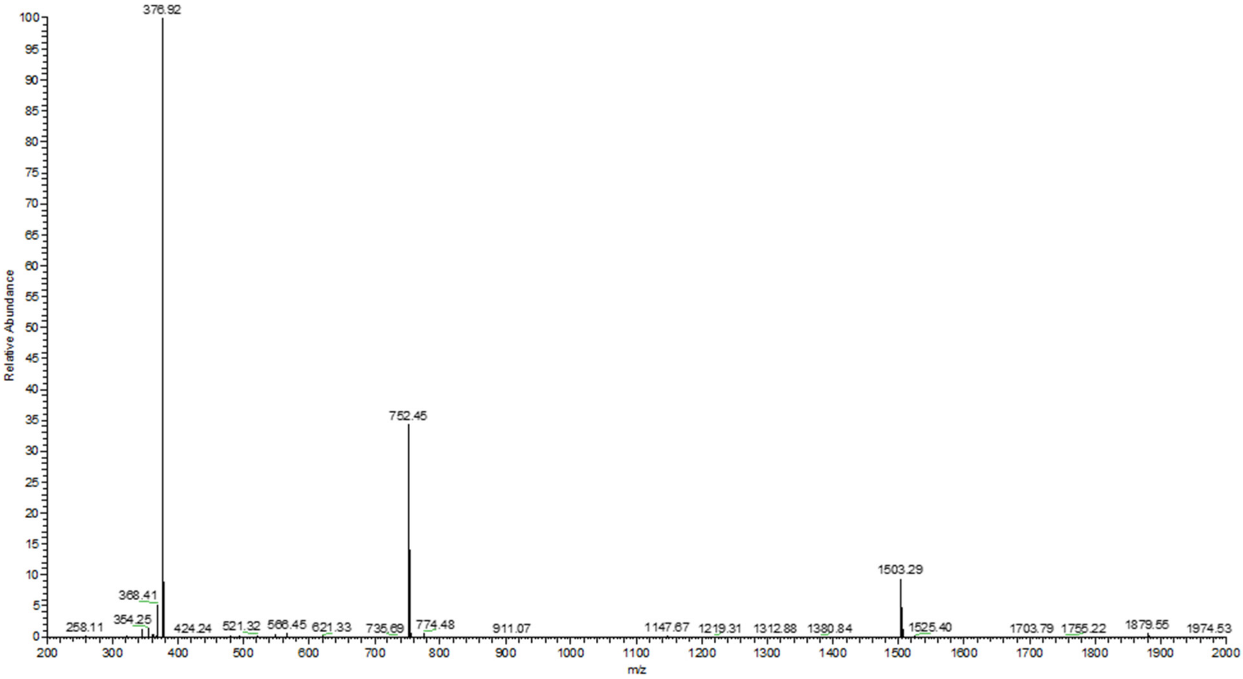

376.92
752.45
1503.29
368.41
354.25
258.11
424.24
521.32
566.45
621.33
735.69
774.48
911.07
1147.67
1219.31
1312.88
1380.84
1525.40
1703.79
1755.22
1879.55
1974.53
Relative Abundance
m/z

WYKYA

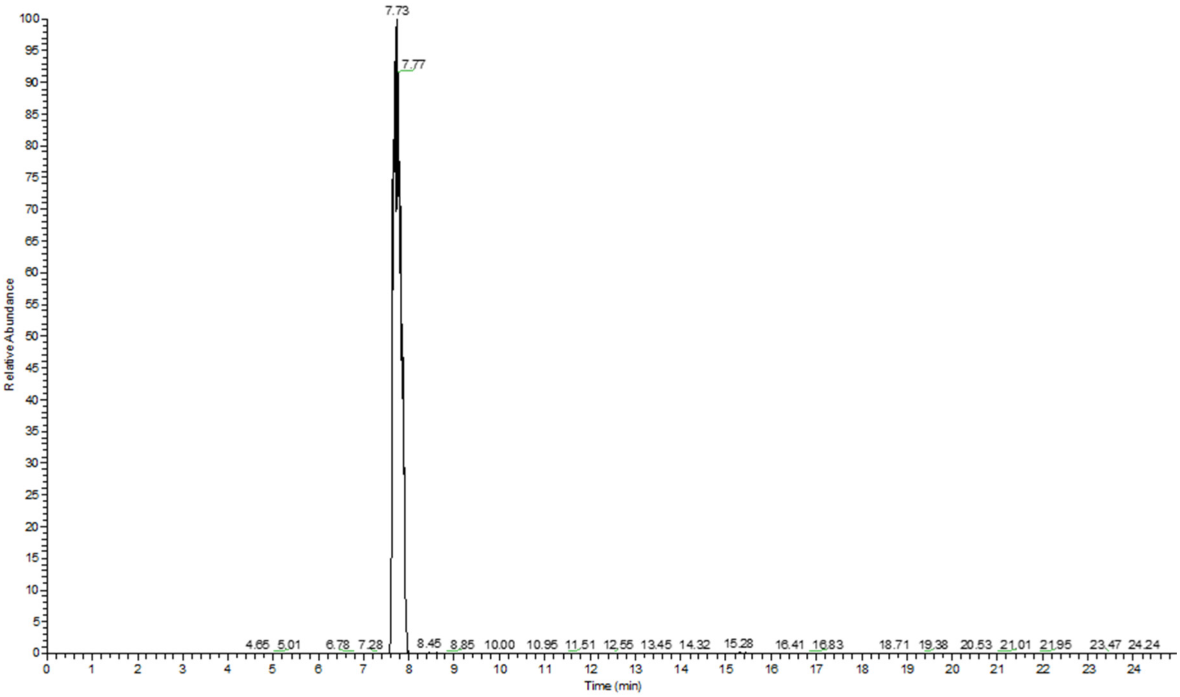
7.73
7.77
4.65
5.01
6.78
7.28
8.45
8.85
10.00
10.95
11.51
12.55
13.45
14.32
15.28
16.41
16.83
18.71
19.38
20.53
21.01
21.95
23.47
24.24
Relative Abundance
Time (min)

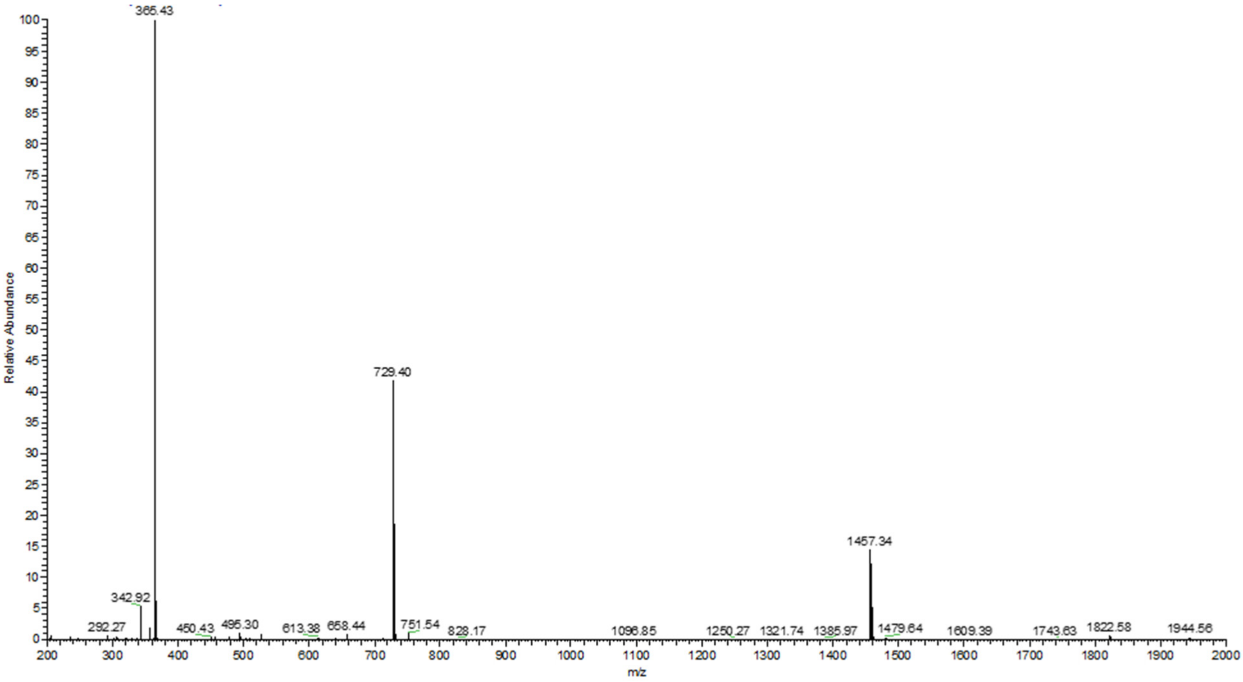
365.43
729.40
1457.34
342.92
292.27
450.43
495.30
613.38
658.44
751.54
828.17
1096.85
1250.27
1321.74
1385.97
1479.64
1609.39
1743.63
1822.58
1944.56
Relative Abundance
m/z

wYKYW

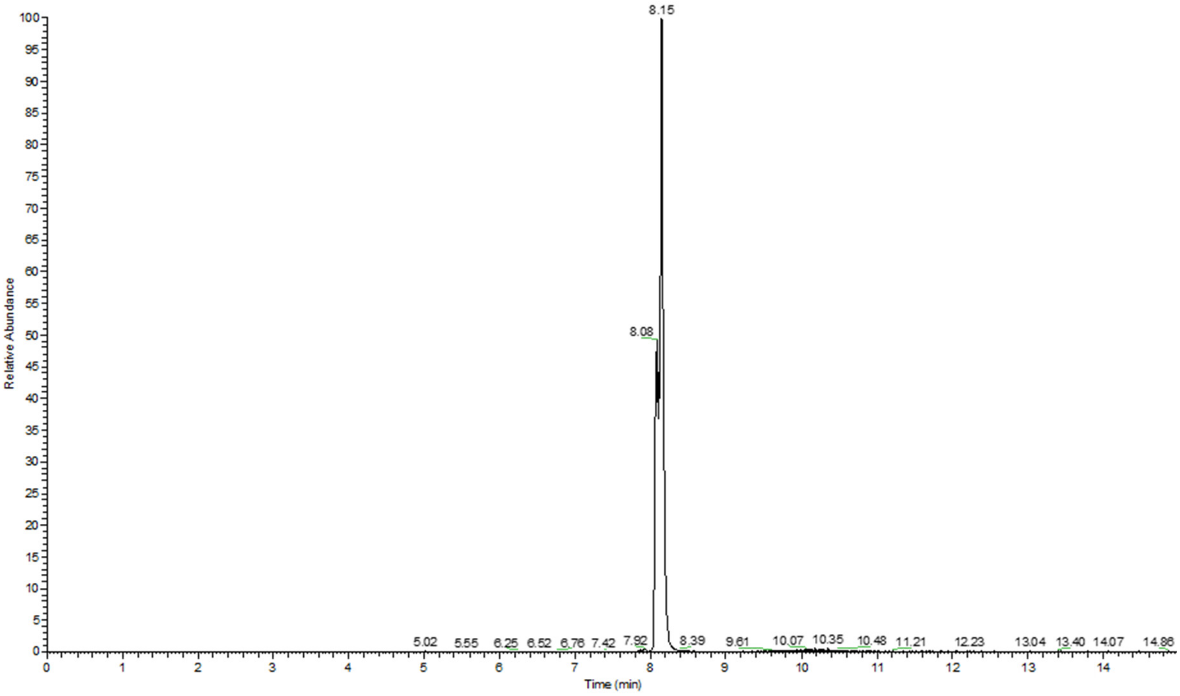
Relative Abundance
Time (min)
8.15
8.08

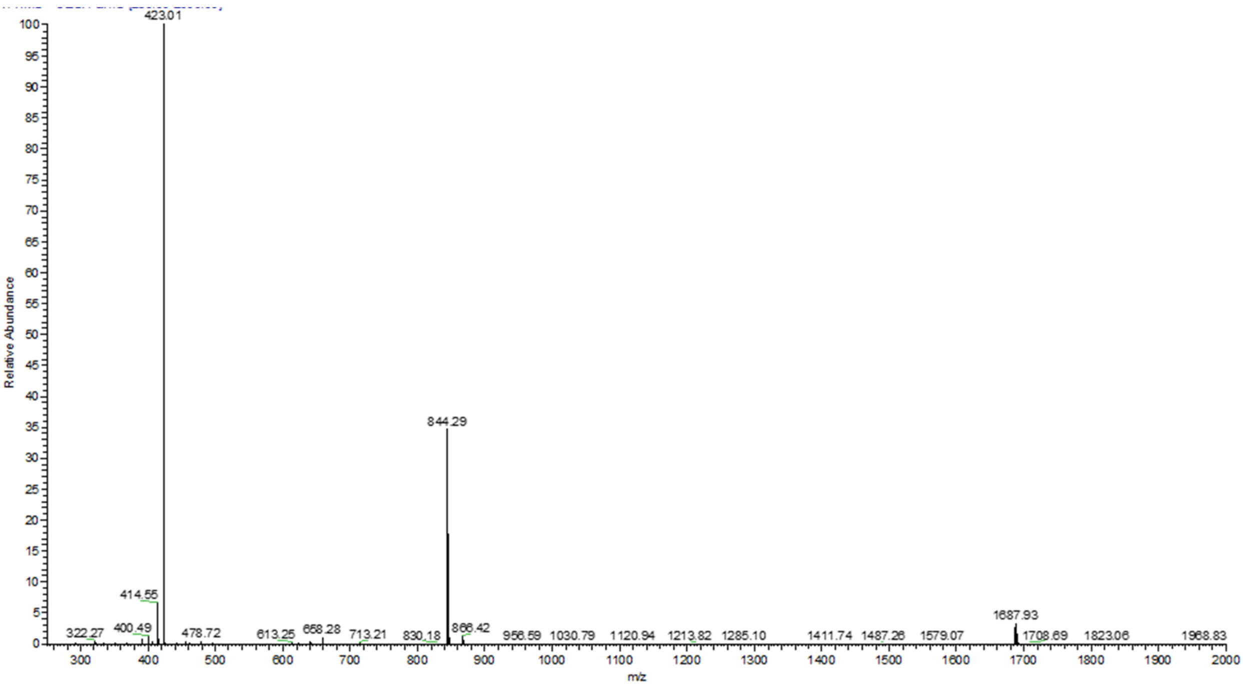
Relative Abundance
m/z
423.01
844.29
414.55
1687.93

WyKYW

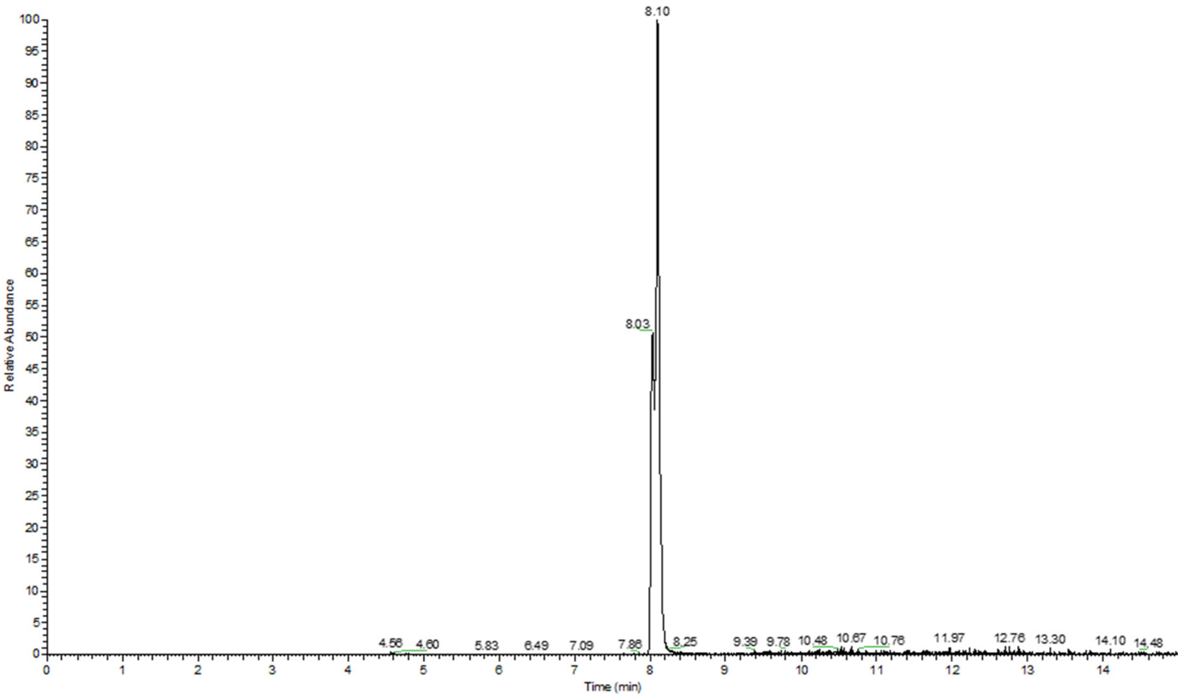
8.10
8.03
4.56
4.60
5.83
6.49
7.09
7.86
8.25
9.39
9.78
10.48
10.67
10.76
11.97
12.76
13.30
14.10
14.48
Relative Abundance
Time (min)

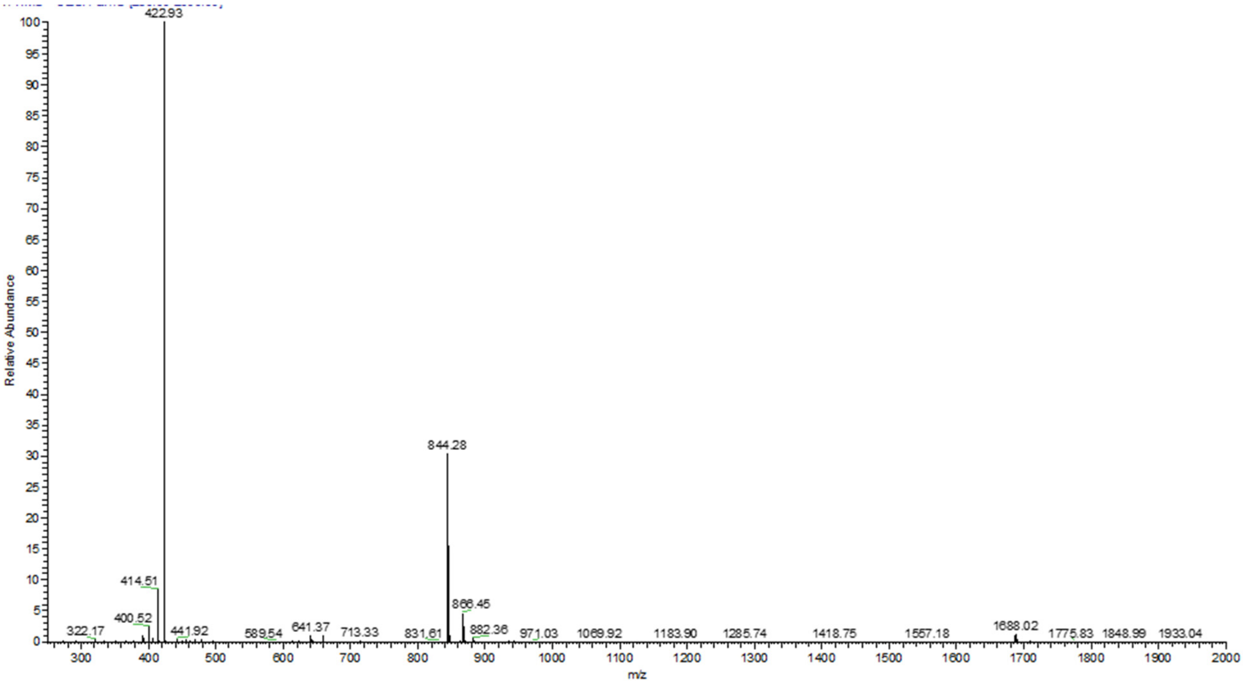
422.93
844.28
414.51
400.52
322.17
441.92
589.54
641.37
713.33
831.61
866.45
882.36
971.03
1069.92
1183.90
1285.74
1418.75
1557.18
1688.02
1775.83
1848.99
1933.04
Relative Abundance
m/z

WYkYW

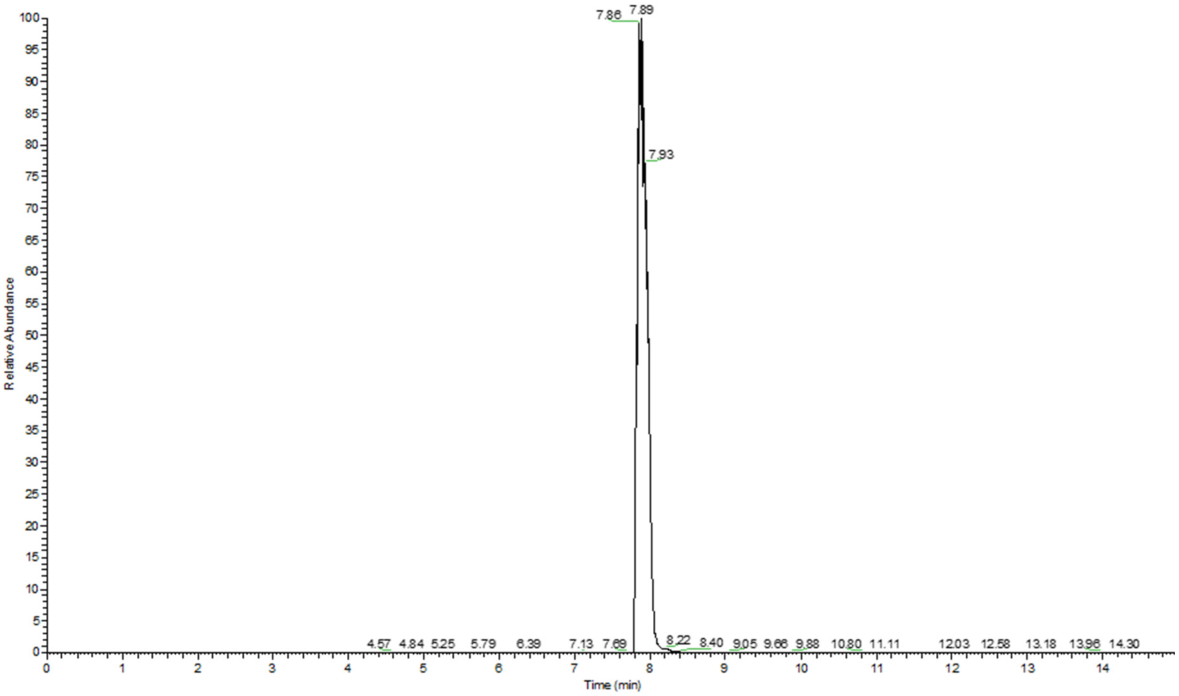

7.86
7.89
7.93
8.22
8.40
Relative Abundance
Time (min)

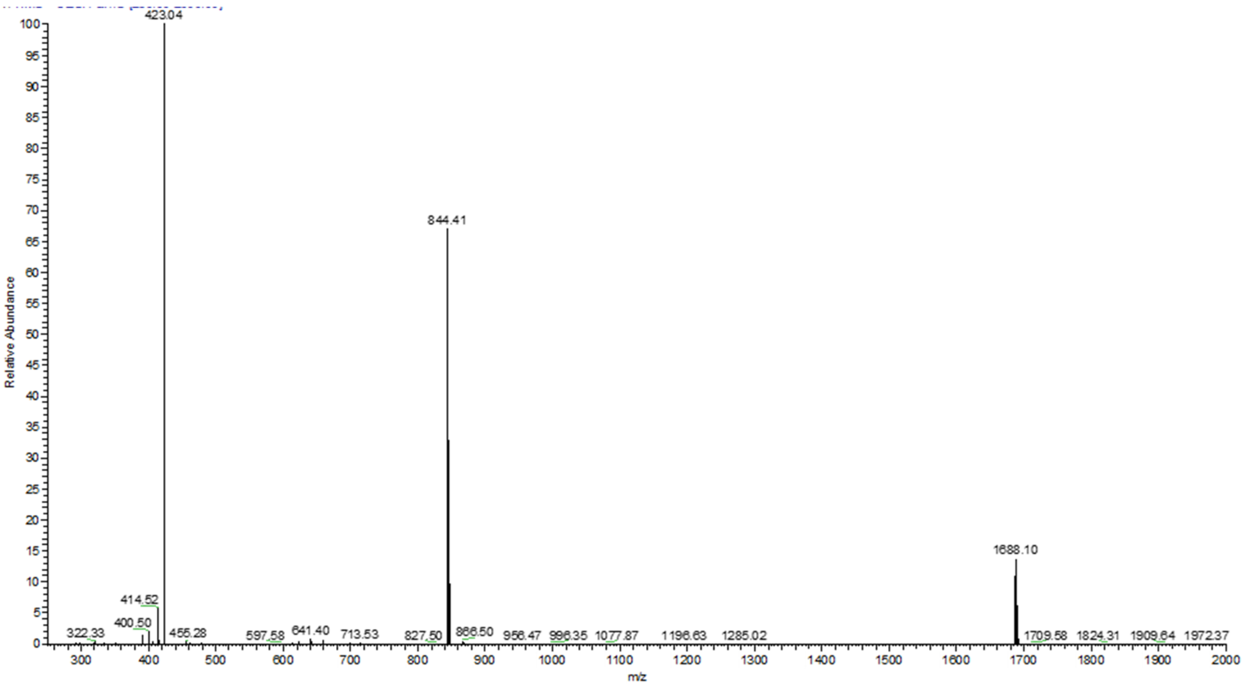

423.04
844.41
1688.10
414.52
400.50
Relative Abundance
m/z

WYKyW

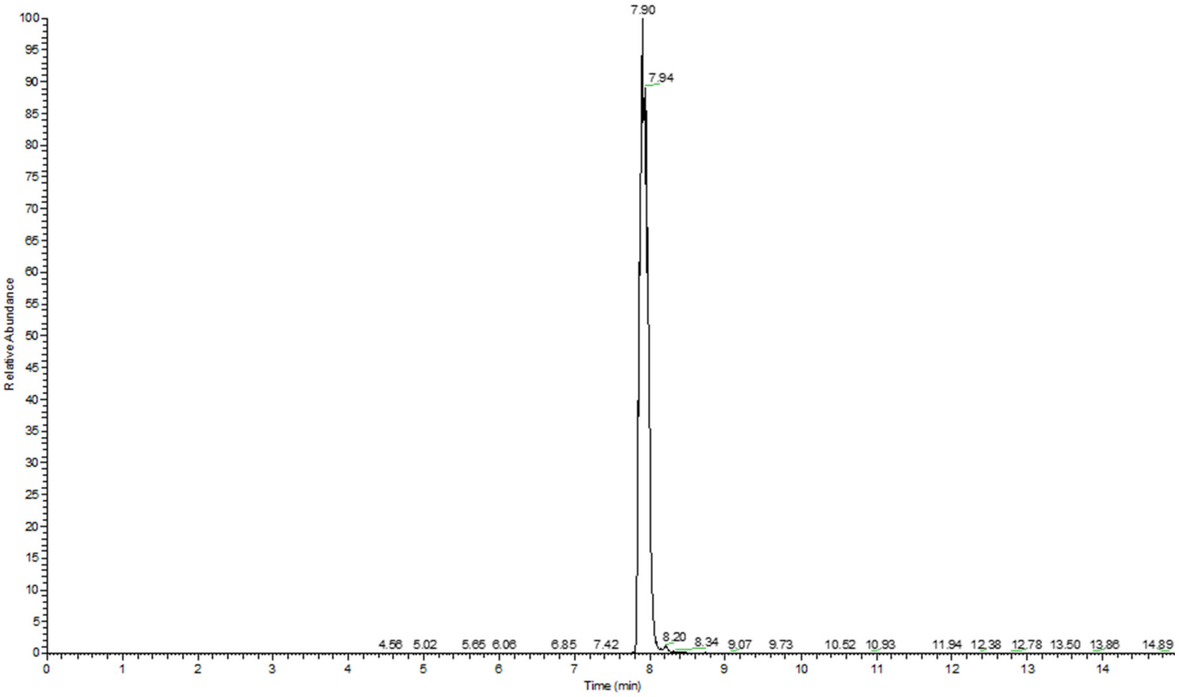
7.90
7.94
8.20
8.34
4.56 5.02 5.65 6.06 6.85 7.42 9.07 9.73 10.52 10.93 11.94 12.38 12.78 13.50 13.86 14.89
Relative Abundance
Time (min)

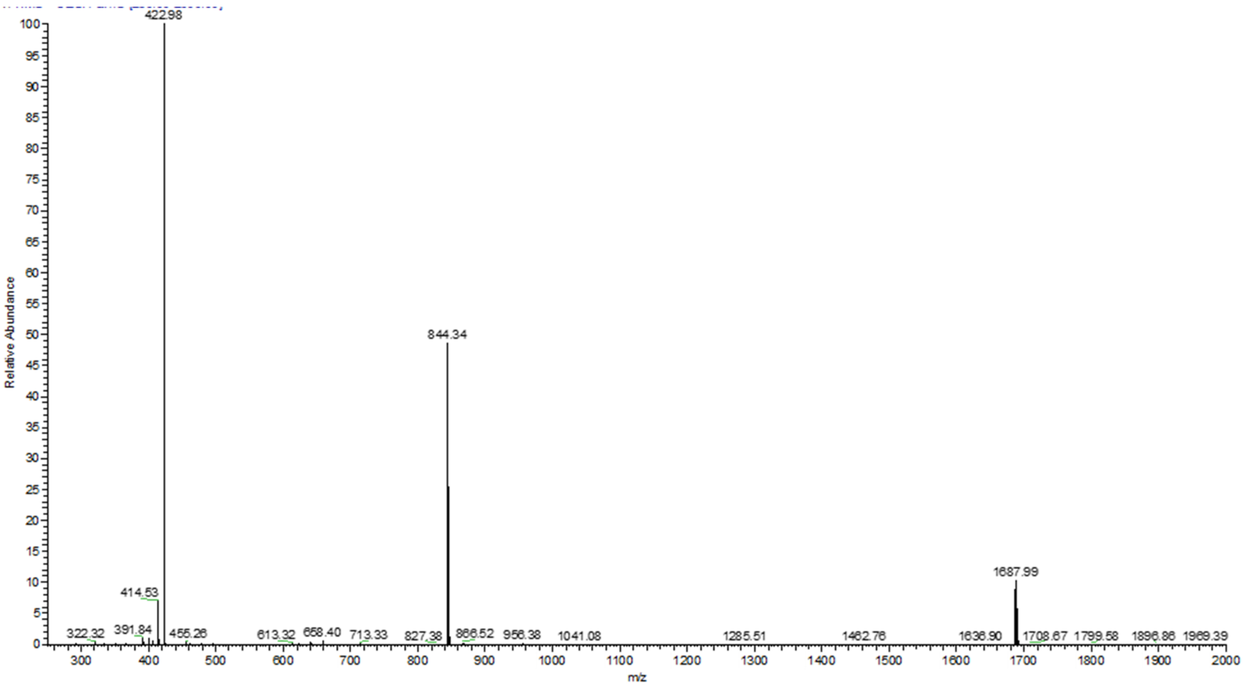
422.98
844.34
1687.99
414.53
322.32 391.84 455.26 613.32 658.40 713.33 827.38 866.52 956.38 1041.08 1285.51 1462.76 1636.90 1708.67 1799.58 1896.86 1969.39
Relative Abundance
m/z

WYKYw

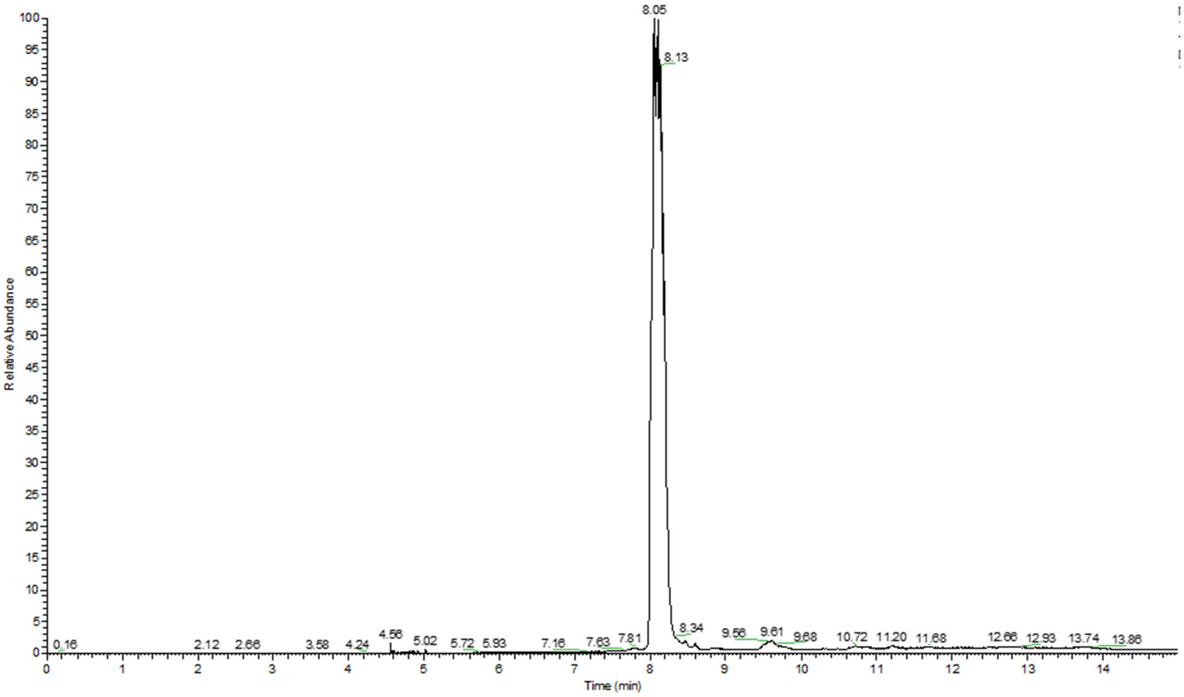
8.05
8.13
8.34
9.56
9.61
9.68
10.72
11.20
11.68
12.66
12.93
13.74
13.86
0.16
2.12
2.66
3.58
4.24
4.56
5.02
5.72
5.93
7.16
7.63
7.81
Relative Abundance
Time (min)

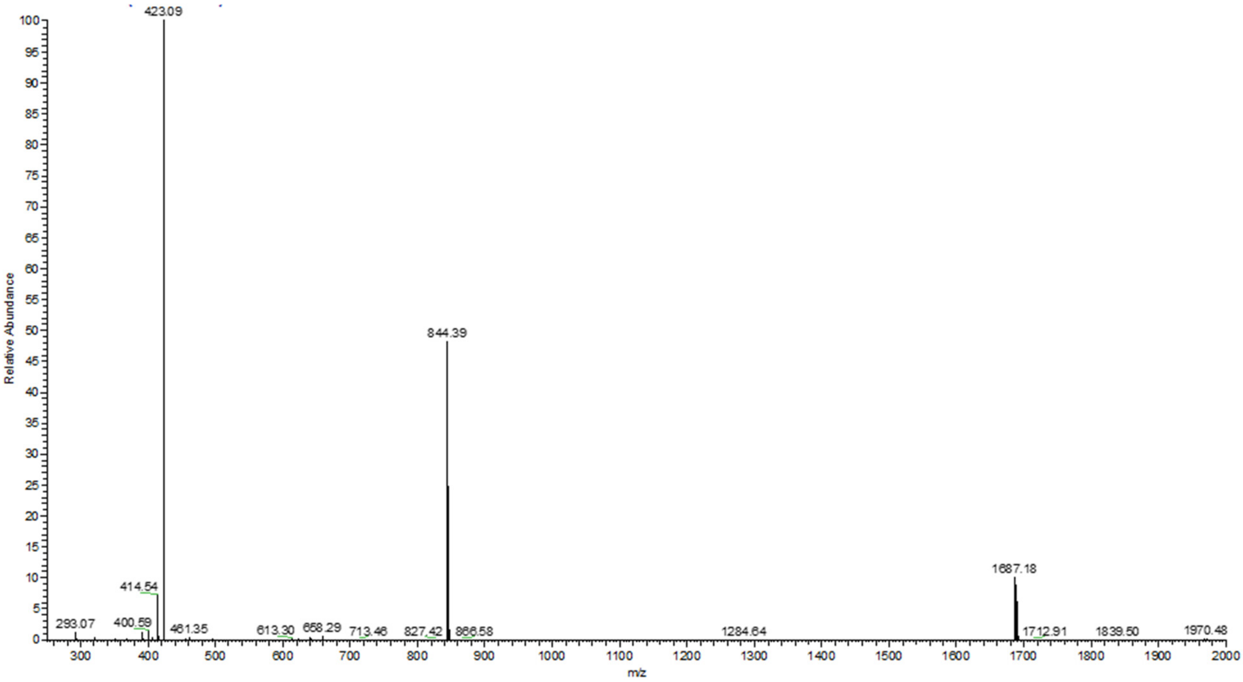
423.09
844.39
1687.18
414.54
293.07
400.59
461.35
613.30
658.29
713.46
827.42
866.58
1284.64
1712.91
1839.50
1970.48
Relative Abundance
m/z

WAKAW

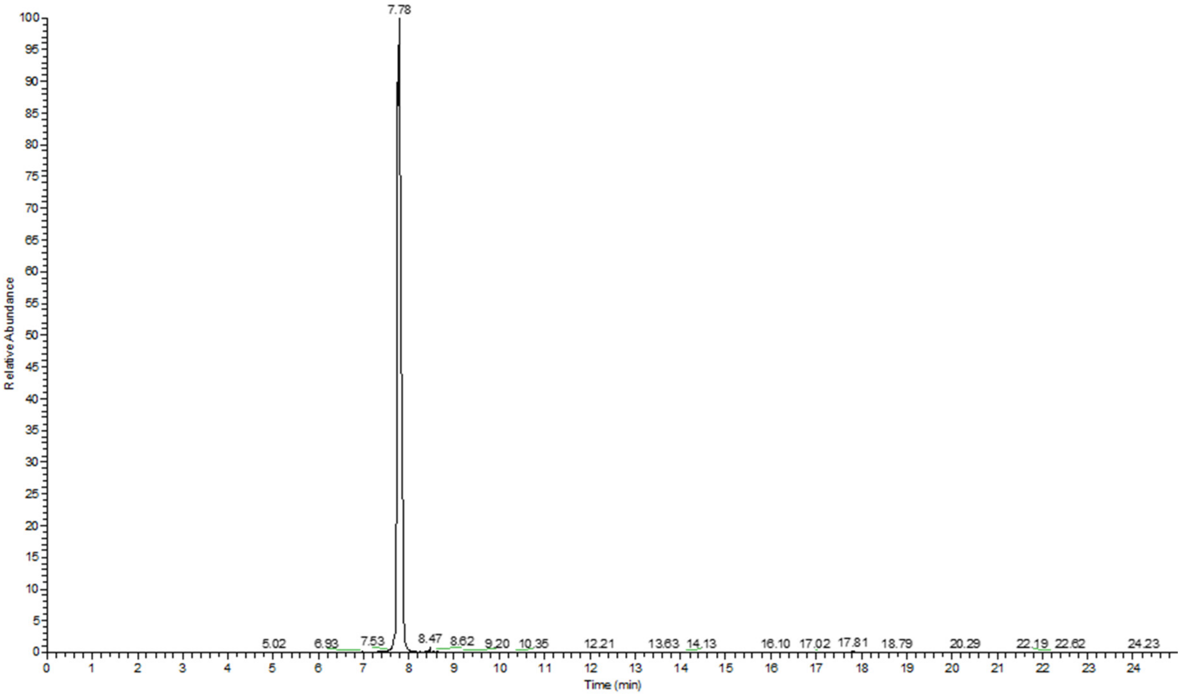
7.78
Relative Abundance
5.02
6.93
7.53
8.47
8.62
9.20
10.35
12.21
13.63
14.13
16.10
17.02
17.81
18.79
20.29
22.19
22.62
24.23
Time (min)

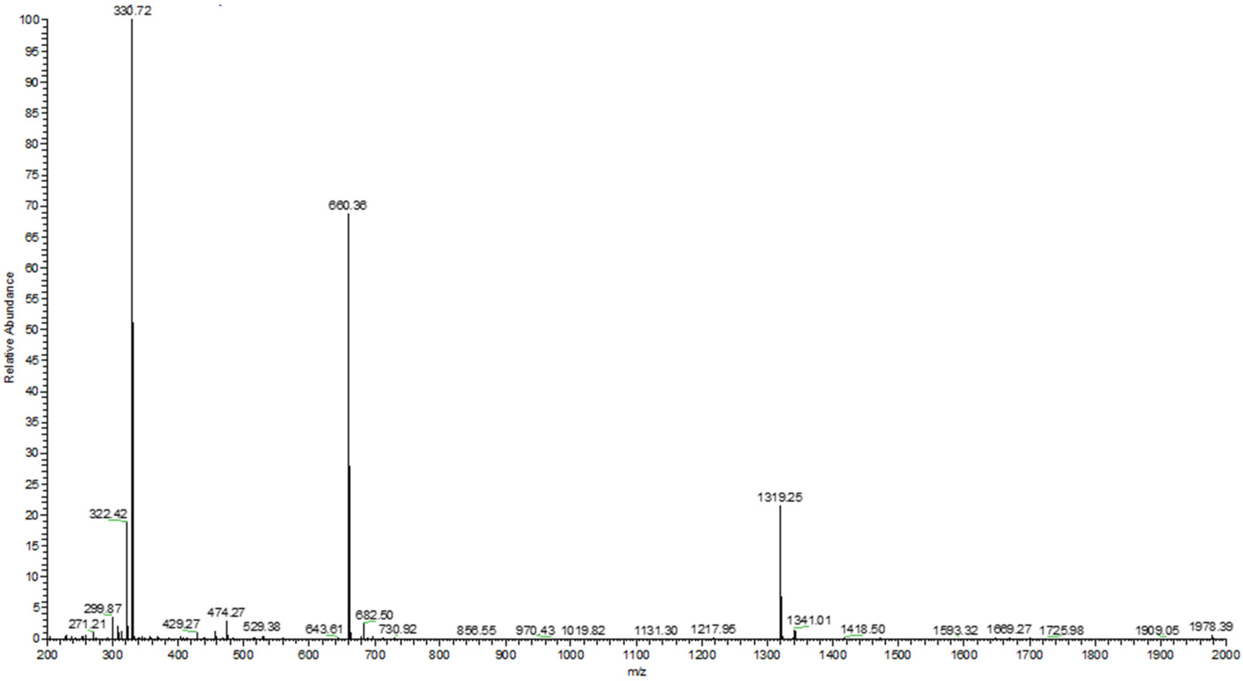
330.72
Relative Abundance
660.36
322.42
1319.25
299.87
271.21
429.27
474.27
529.38
643.61
682.50
730.92
856.55
970.43
1019.82
1131.30
1217.95
1341.01
1418.50
1593.32
1669.27
1725.98
1909.05
1978.39
m/z

AYKYA

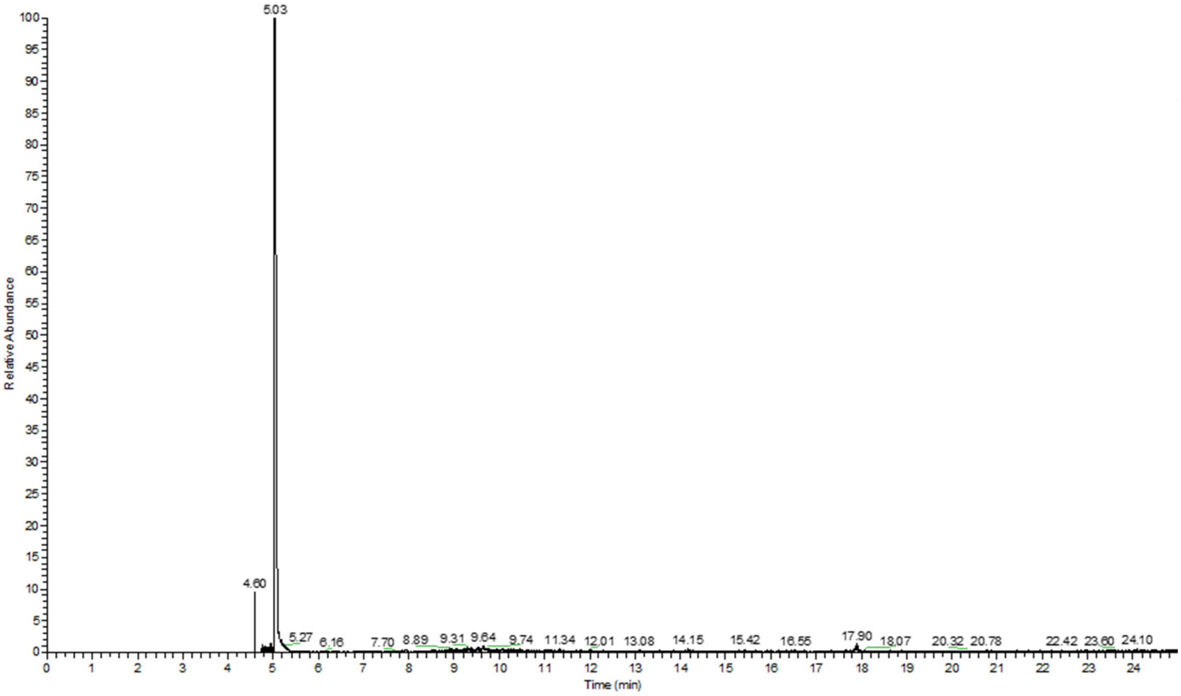
5.03
4.60
5.27
6.16
7.70
8.89
9.31
9.64
9.74
11.34
12.01
13.08
14.15
15.42
16.55
17.90
18.07
20.32
20.78
22.42
23.60
24.10
Relative Abundance
Time (min)

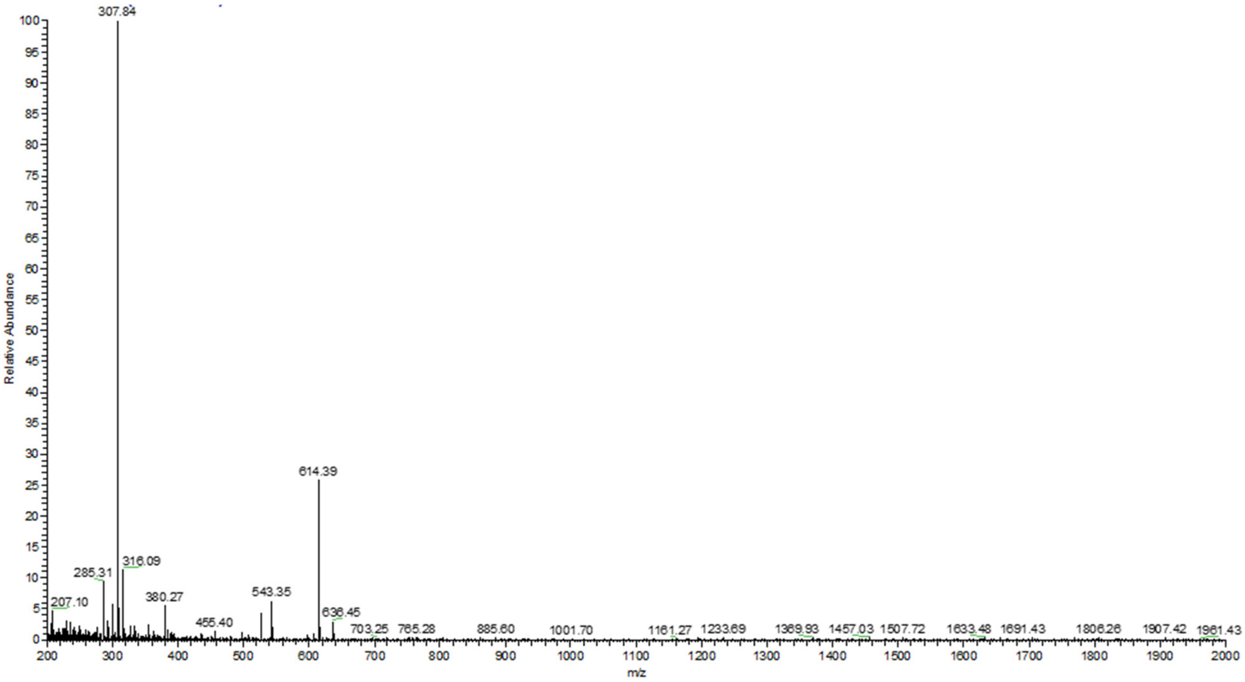
307.84
614.39
285.31
316.09
207.10
380.27
543.35
455.40
636.45
703.25
765.28
885.60
1001.70
1161.27
1233.69
1369.93
1457.03
1507.72
1633.48
1691.43
1806.26
1907.42
1961.43
Relative Abundance
m/z

Biotin-Penetratin-GG-W$^{\beta}$YKYW (K(Biotin)-RQIKIWFQNRRMKWKKGGW$^{\beta}$YKYW-NH$_2$)

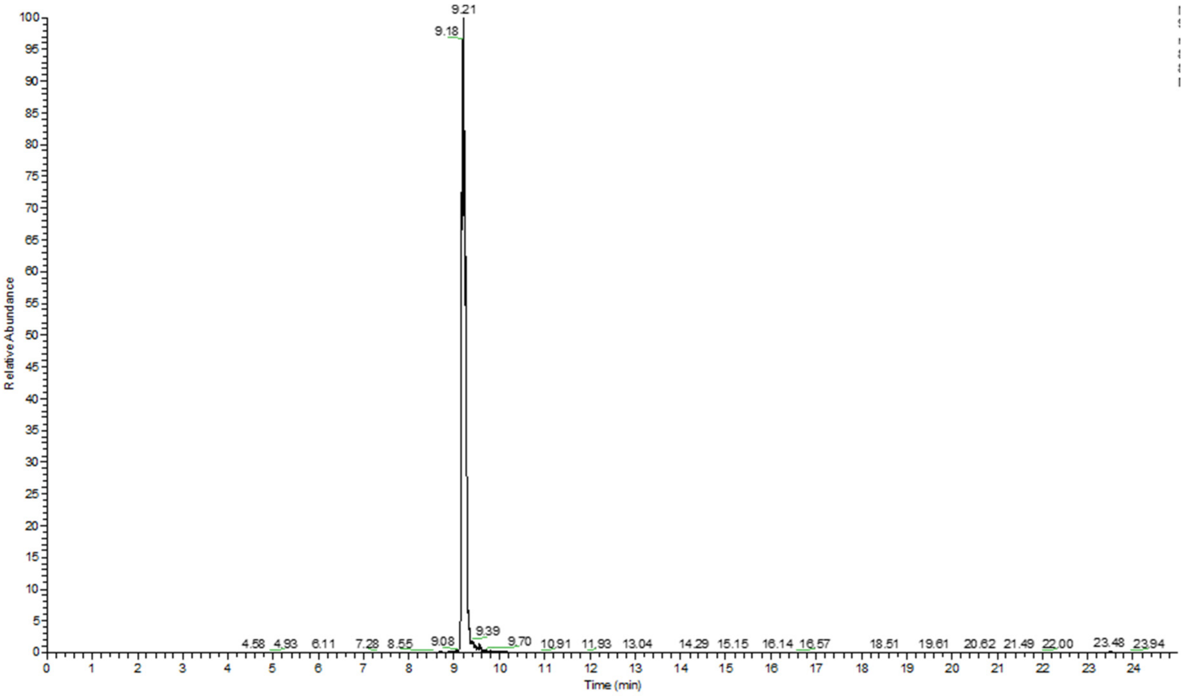

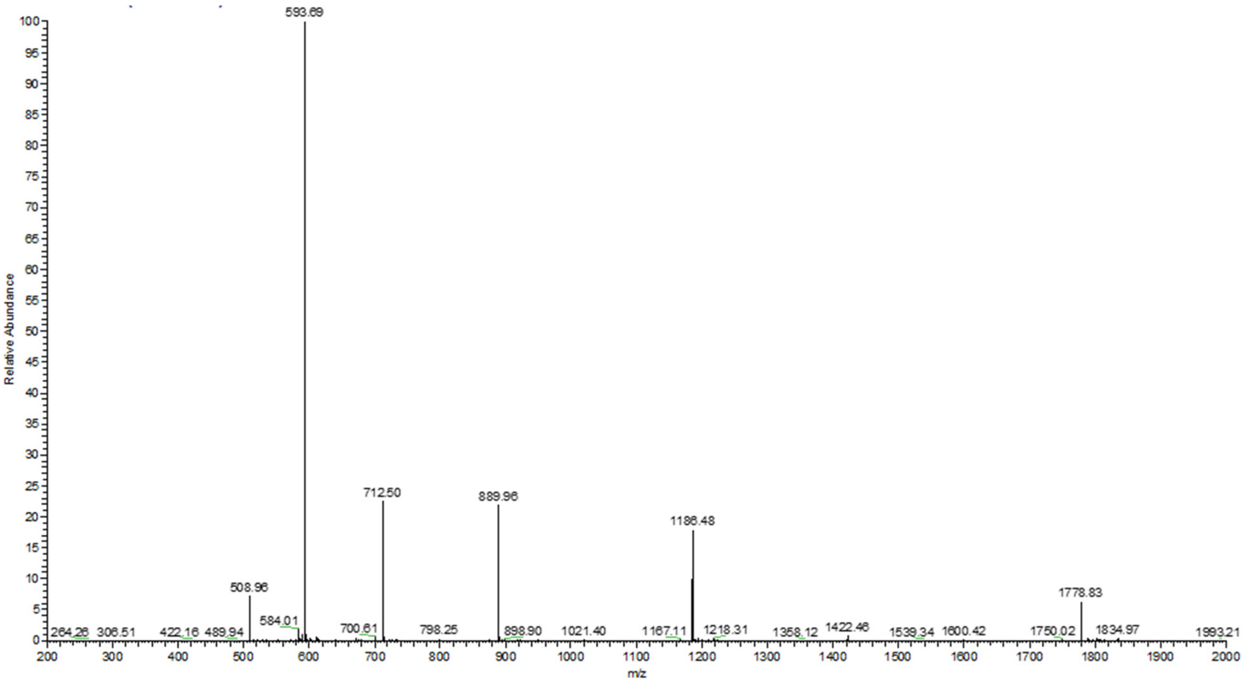

Biotin-Penetratin-GG-WY$^{\beta}$KYW (K(Biotin)-RQIKIWFQNRRMKWKKGGWY$^{\beta}$KYW-NH$_2$)

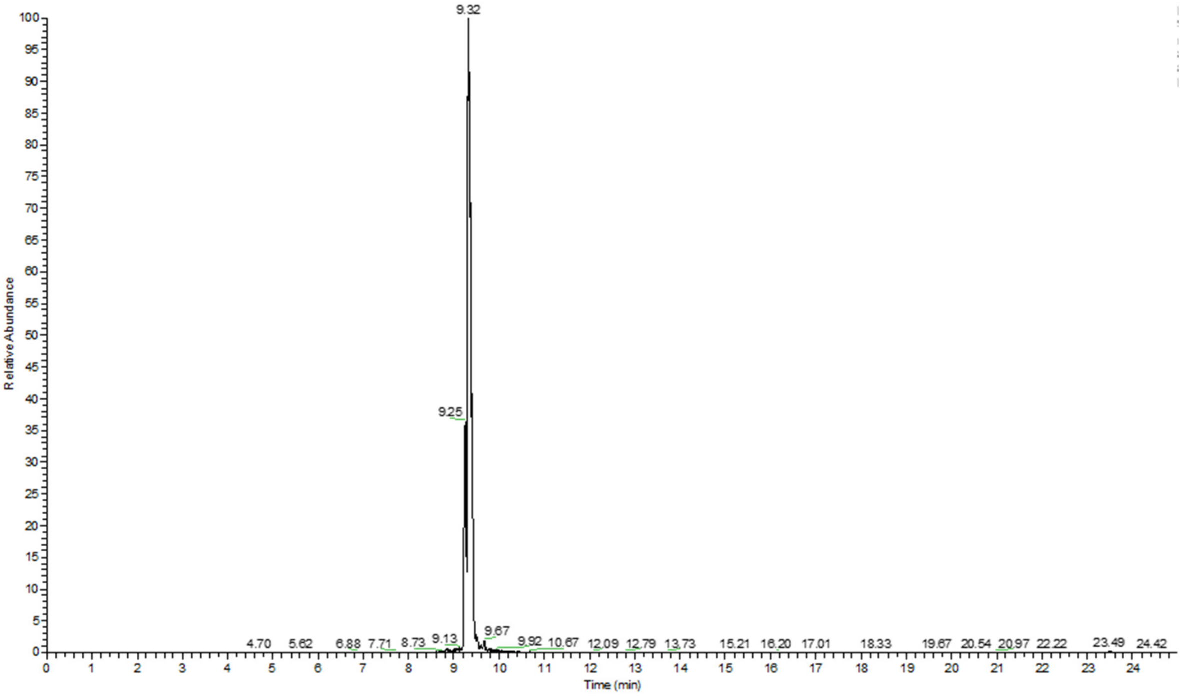

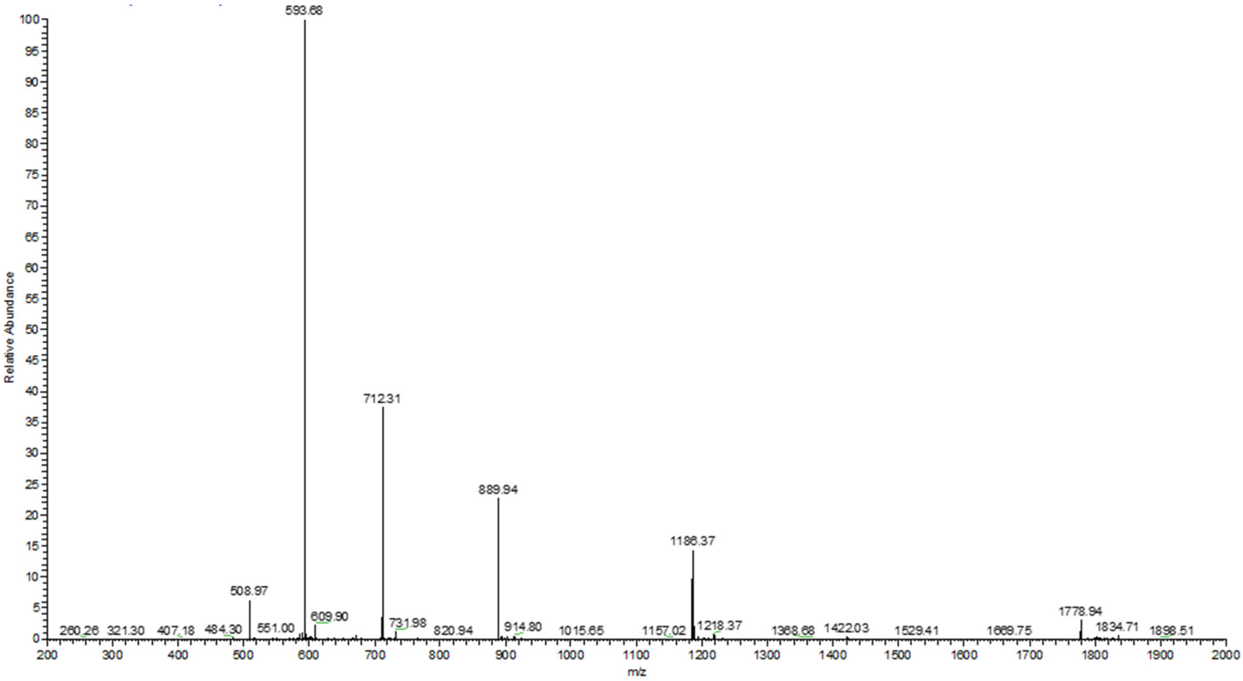

Biotin-Penetratin-GG-WYK^{β}YW (K(Biotin)-RQIKIWFQNRRMKWKKGGWYK^{β}YW-NH_2)

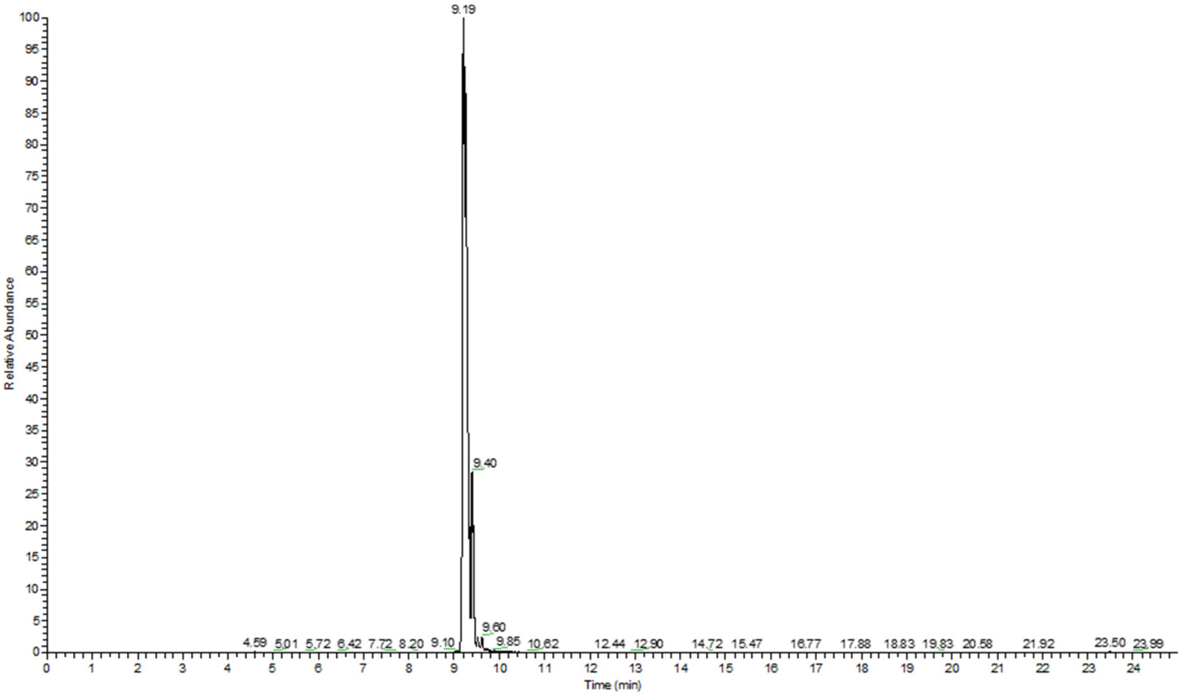

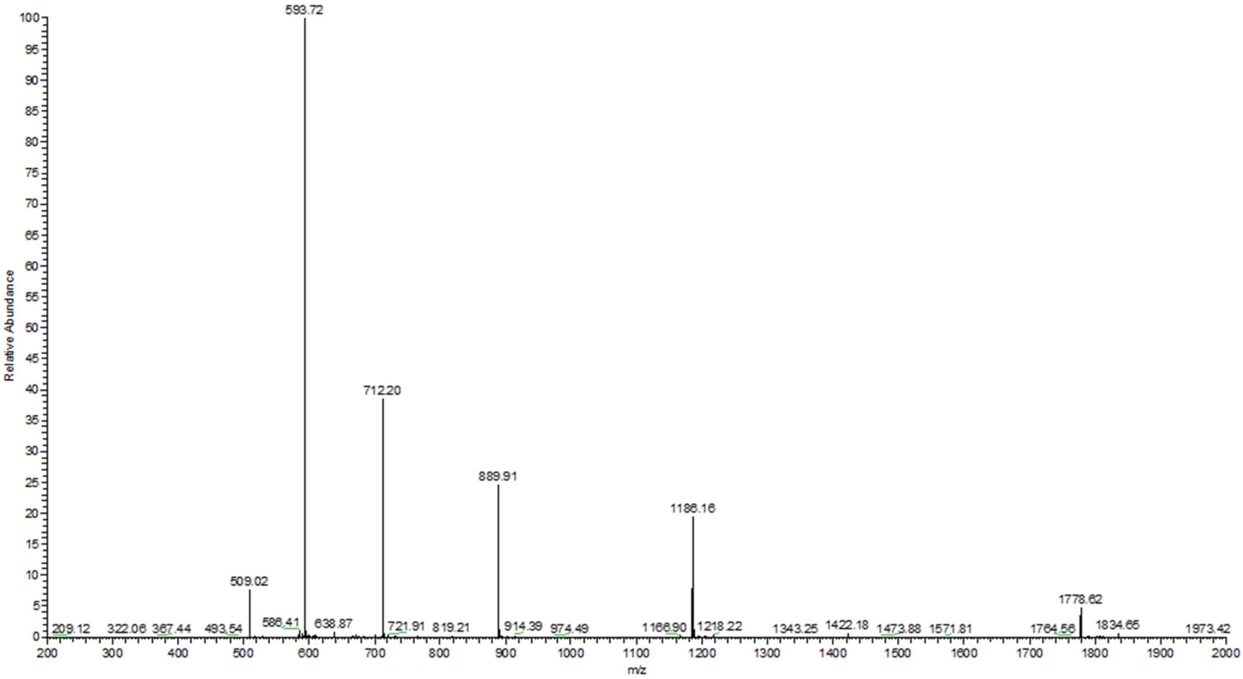

Biotin-Penetratin-GG-WYKY$^{\beta}$W (K(Biotin)-RQIKIWFQNRRMKWKKGGWYKY$^{\beta}$W-NH_2)

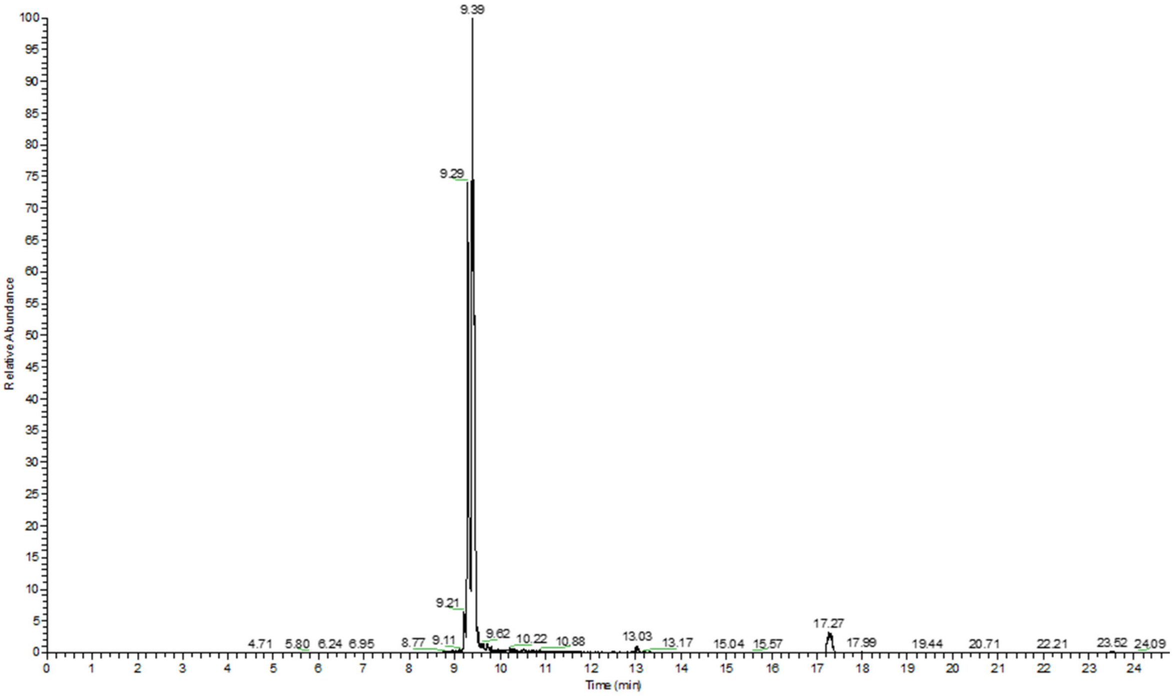

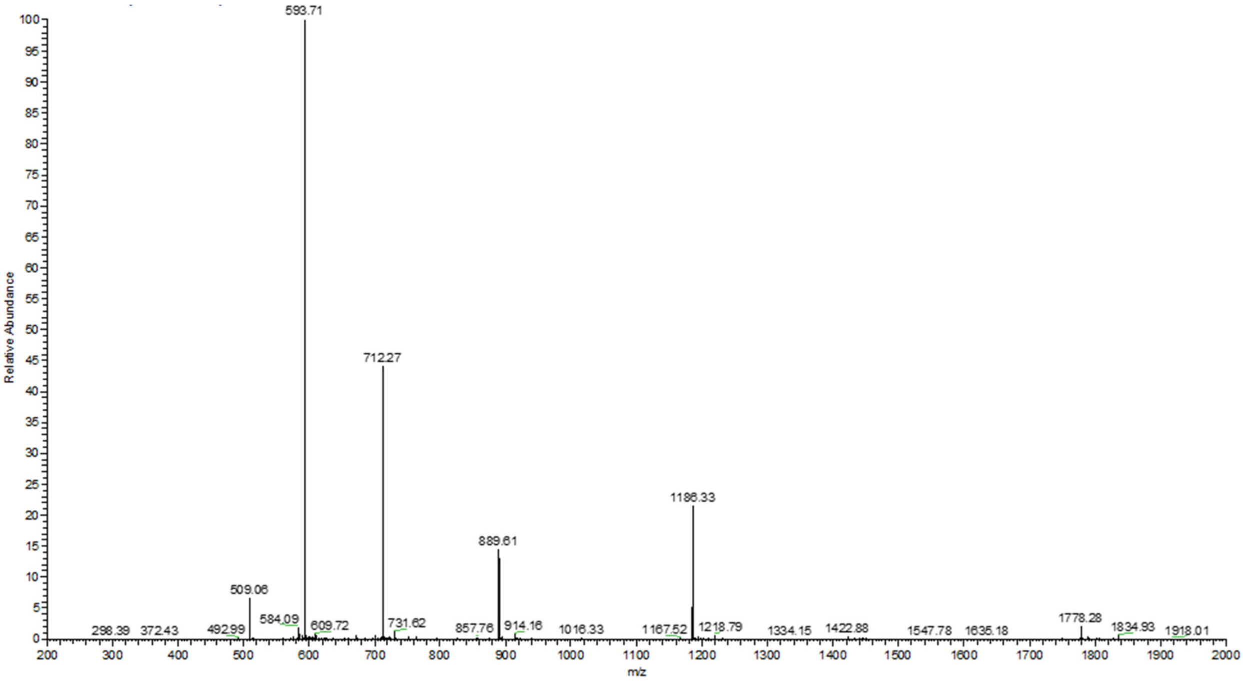

Biotin-Penetratin-GG-WYKYW$^{\beta}$ (K(Biotin)-RQIKIWFQNRRMKWKKGGWYKYW$^{\beta}$-NH$_2$)

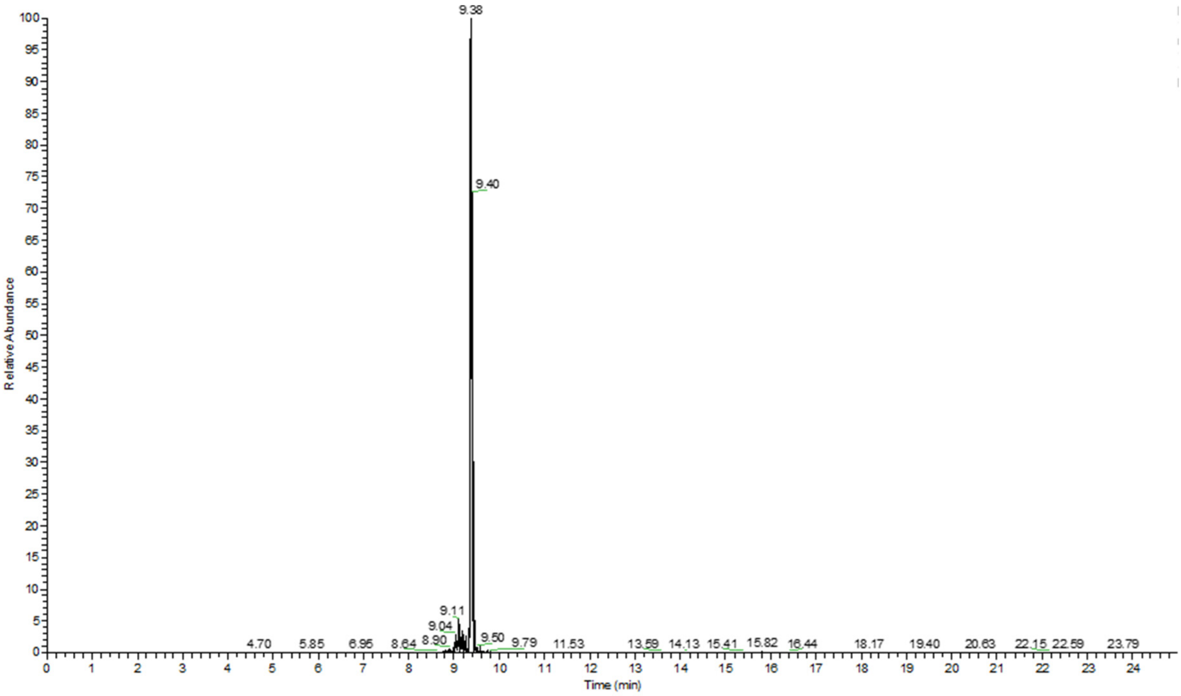

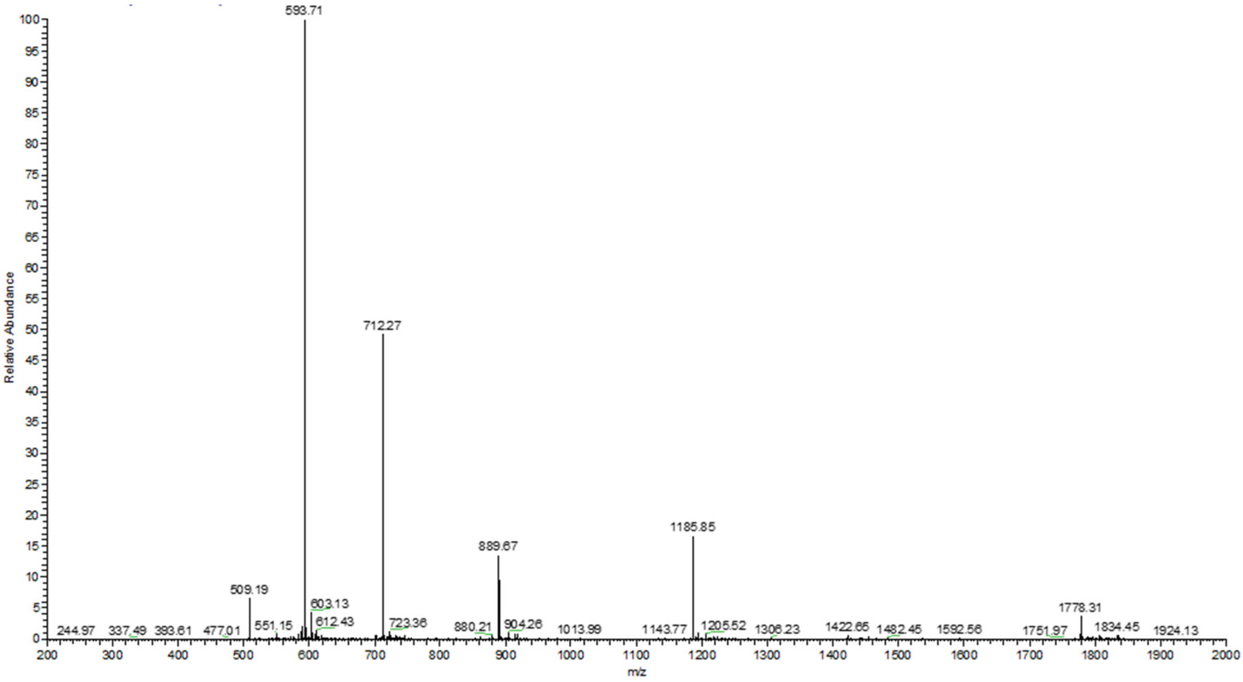

Biotin-Penetratin-GG-WYKYW (K(Biotin)-RQIKIWFQNRRMKWKKGGWYKYW-NH$_2$)

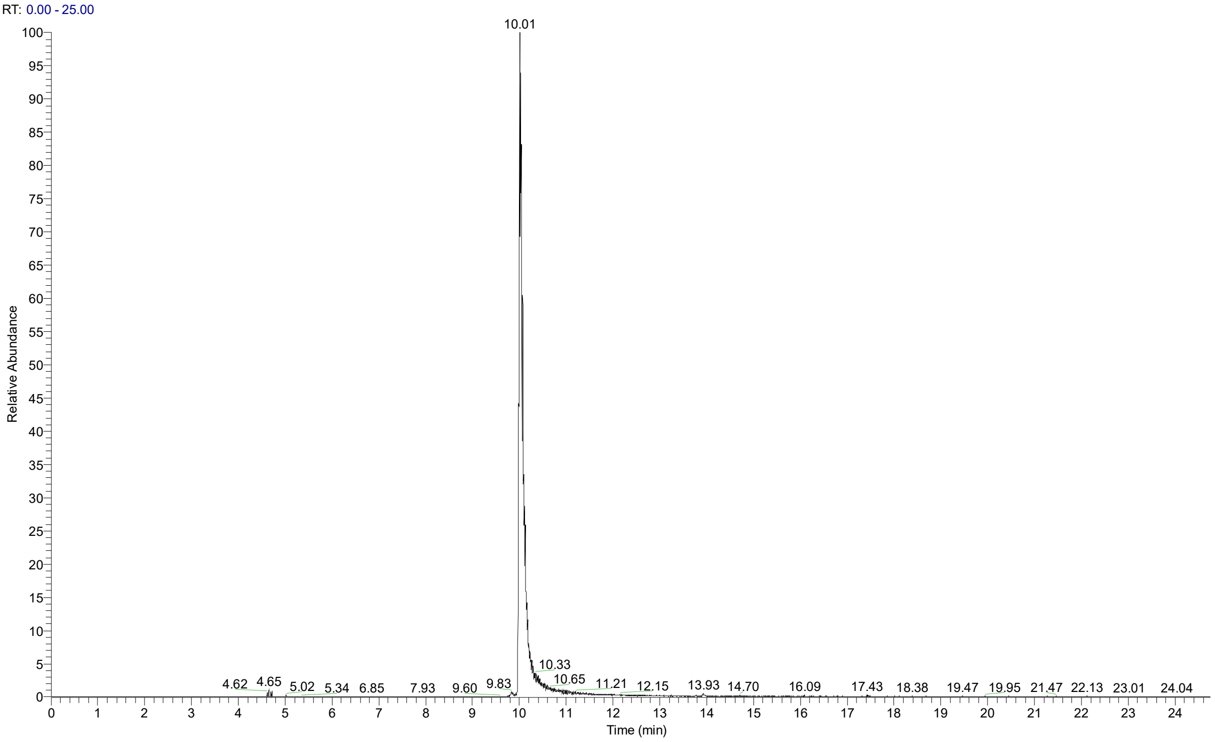

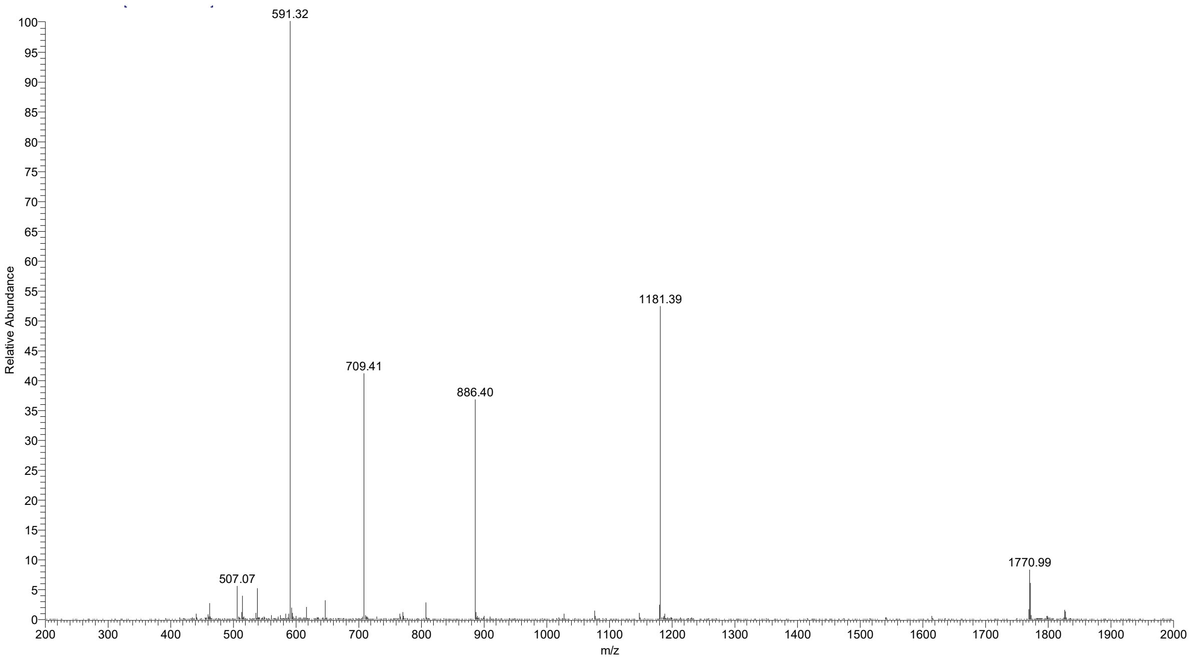

Supplement: Supplementary file 1 [file pharmaceutics-14-00580-s001.zip › pharmaceutics-1594486-supplementary.pdf]
